# The potential impact of urine-LAM diagnostics on tuberculosis incidence and mortality: A modelling analysis

**DOI:** 10.1371/journal.pmed.1003466

**Published:** 2020-12-11

**Authors:** Saskia Ricks, Claudia M. Denkinger, Samuel G. Schumacher, Timothy B. Hallett, Nimalan Arinaminpathy

**Affiliations:** 1 MRC Centre for Global Infectious Disease Analysis, Imperial College London, London, United Kingdom; 2 Center of Infectious Disease, University of Heidelberg, Heidelberg, Germany; 3 Foundation for Innovative New Diagnostics, Geneva, Switzerland; Centers for Disease Control, UNITED STATES

## Abstract

**Background:**

Lateral flow urine lipoarabinomannan (LAM) tests could offer important new opportunities for the early detection of tuberculosis (TB). The currently licensed LAM test, Alere Determine TB LAM Ag (‘LF-LAM’), performs best in the sickest people living with HIV (PLHIV). However, the technology continues to improve, with newer LAM tests, such as Fujifilm SILVAMP TB LAM (‘SILVAMP-LAM’) showing improved sensitivity, including amongst HIV-negative patients. It is important to anticipate the epidemiological impact that current and future LAM tests may have on TB incidence and mortality.

**Methods and findings:**

Concentrating on South Africa, we examined the impact that widening LAM test eligibility would have on TB incidence and mortality. We developed a mathematical model of TB transmission to project the impact of LAM tests, distinguishing ‘current’ tests (with sensitivity consistent with LF-LAM), from hypothetical ‘future’ tests (having sensitivity consistent with SILVAMP-LAM). We modelled the impact of both tests, assuming full adoption of the 2019 WHO guidelines for the use of these tests amongst those receiving HIV care. We also simulated the hypothetical deployment of future LAM tests for all people presenting to care with TB symptoms, not restricted to PLHIV. Our model projects that 2,700,000 (95% credible interval [CrI] 2,000,000–3,600,000) and 420,000 (95% CrI 350,000–520,000) cumulative TB incident cases and deaths, respectively, would occur between 2020 and 2035 if the status quo is maintained. Relative to this comparator, current and future LAM tests would respectively avert 54 (95% CrI 33–86) and 90 (95% CrI 55–145) TB deaths amongst inpatients between 2020 and 2035, i.e., reductions of 5% (95% CrI 4%–6%) and 9% (95% CrI 7%–11%) in inpatient TB mortality. This impact in absolute deaths averted doubles if testing is expanded to include outpatients, yet remains <1% of country-level TB deaths. Similar patterns apply to incidence results. However, deploying a future LAM test for all people presenting to care with TB symptoms would avert 470,000 (95% CrI 220,000–870,000) incident TB cases (18% reduction, 95% CrI 9%–29%) and 120,000 (95% CrI 69,000–210,000) deaths (30% reduction, 95% CrI 18%–44%) between 2020 and 2035. Notably, this increase in impact arises largely from diagnosis of TB amongst those with HIV who are not yet in HIV care, and who would thus be ineligible for a LAM test under current guidelines. Qualitatively similar results apply under an alternative comparator assuming expanded use of GeneXpert MTB/RIF (‘Xpert’) for TB diagnosis. Sensitivity analysis demonstrates qualitatively similar results in a setting like Kenya, which also has a generalised HIV epidemic, but a lower burden of HIV/TB coinfection. Amongst limitations of this analysis, we do not address the cost or cost-effectiveness of future tests. Our model neglects drug resistance and focuses on the country-level epidemic, thus ignoring subnational variations in HIV and TB burden.

**Conclusions:**

These results suggest that LAM tests could have an important effect in averting TB deaths amongst PLHIV with advanced disease. However, achieving population-level impact on the TB epidemic, even in high-HIV-burden settings, will require future LAM tests to have sufficient performance to be deployed more broadly than in HIV care.

## Abstract 2

Saskia Ricks and colleagues model the impact of urine-LAM diagnostics for reducing tuberculosis incidence, across different implementation scenarios.

## Author summary

**Why was this study done?:**

- Although tuberculosis (TB) is often a disease of the lungs, new tests are being developed that can diagnose TB using urine samples alone, and that are simple enough to be used with minimal training.
- Current urine-based lipoarabinomannan (LAM) tests only perform well in the sickest patients, especially those infected by both TB and HIV. As a result, current WHO guidelines only recommend the use of these tests in specific patient groups.
- Recent years have seen the emergence of newer LAM tests with improved performance, even amongst patients without HIV infection.
- There is a need to understand the impact that these LAM tests may have on the TB epidemic across different implementation scenarios.

**What did the researchers do and find?:**

- We developed a mathematical model that captures TB and HIV dynamics in South Africa, a country where 59% of TB cases are coinfected with HIV.
- We modelled the adoption of 2019 WHO guidelines for the use of the currently licensed LAM test amongst those in HIV care. We also modelled a hypothetical scenario where a future LAM test is used outside HIV care, amongst all patients being tested for TB, regardless of HIV status.
- When used only to test for TB amongst HIV patients, LAM testing would avert less than 1% of the total TB cases and deaths in South Africa between 2020 and 2035. However, using a future LAM test to test for TB amongst all people with symptoms, regardless of their HIV status, could avert 30% of TB deaths and 18% of TB cases between 2020 and 2035.

**What do these findings mean?:**

- LAM tests can save lives amongst the sickest TB patients. However, to reduce a country’s TB epidemic, future LAM tests will need to have sufficient performance to be deployed more broadly than in HIV care.
- Future work is needed to determine the cost-effectiveness of deployment as new LAM tests are developed.

## Introduction

There is a pressing need for new approaches to diagnose tuberculosis (TB), in order to accelerate current slow declines in TB incidence and mortality [1,2]. In TB diagnosis, microbiological confirmation is typically conducted on sputum samples, using either traditional tools such as smear microscopy [3] or, more recently, rapid molecular tests such as GeneXpert MTB/RIF (‘Xpert’) [4,5]. However, sputum-based tests have several limitations: Patients can find it difficult to provide good-quality sputum specimens with the required volume, particularly those living with advanced HIV disease [6]. Additionally, sputum-based tests cannot detect extrapulmonary TB in the absence of pulmonary involvement. For these reasons, there has been increasing interest in new non-sputum-based diagnostic tools [7]. In particular, urine-based tests aim to detect the mycobacterium lipoarabinomannan (LAM) antigen, part of the outer cell wall of mycobacteria [6,8–10]. It is less invasive to collect urine than sputum samples in clinical settings, with lower infection risk to healthcare workers. Moreover, urine-based tests can detect extrapulmonary TB [11]. Alere Determine TB LAM Ag (‘LF-LAM’) is the only commercially available urine-based diagnostic for TB [8], a lateral flow LAM assay that is inexpensive and simple enough to be used as a point-of-care test.

However, LF-LAM performs reasonably well only in patients with advanced HIV disease [12]. Consequently, WHO guidelines have restricted the use of LF-LAM to defined patient subgroups, as listed in Table 1 [13]. Clinical trials show that LF-LAM may have a valuable impact in increasing early treatment and averting TB deaths in these populations [10,12]. The technology continues to improve: A newly developed lateral flow test, Fujifilm SILVAMP TB LAM (‘SILVAMP-LAM’), has shown improved sensitivity for TB compared to LF-LAM, with comparable specificity [9]. Further, a recent study in programmatic conditions demonstrated the potential for SILVAMP-LAM to identify TB amongst HIV-negative patients, showing a sensitivity of approximately 50% in this population [14]. In the same study, the additional positivity from a laboratory-based LAM assay demonstrated the potential sensitivity gains for future LAM tests. Other developments, such as new techniques for concentrating the LAM antigen available in a sample [15], also highlight the potential for continued improvements in the performance of LAM tests.

**Table 1:** WHO 2019 guidelines on the use of lateral flow urine lipoarabinomannan assays for the diagnosis of active tuberculosis (TB) in people living with HIV [13].

Setting	Population among HIV-positive adults, adolescents, and children	Strength of recommendation
Inpatients	With signs and symptoms of TB (pulmonary and/or extrapulmonary)	Strong
	With advanced HIV disease, or who are seriously ill (e.g., with respiratory rate of more than 30/minute, temperature of more than 39°C)	Strong
	Irrespective of signs and symptoms of TB with a CD4 cell count of less than 200 cells/μl	Strong
Outpatients	With signs and symptoms of TB (pulmonary and/or extrapulmonary) or seriously ill	Conditional
	Irrespective of signs and symptoms of TB with a CD4 cell count of less than 100 cells/μl	Conditional

In this context, and as LAM tests continue to improve, it is important to anticipate their potential epidemiological impact, if deployed widely in future. Here, we examined this potential impact using mathematical models of TB transmission, informed by the available evidence for the performance of LF-LAM and SILVAMP-LAM in different healthcare settings. We focused on South Africa, the country with the world’s highest population rates of TB incidence, as well as the highest levels of HIV/TB coinfection. We modelled the potential TB incidence and mortality declines that would arise from the use of currently licensed LAM tests (consistent with LF-LAM) amongst those receiving HIV care, as well as a hypothetical scenario involving the use of potential future LAM tests in routine TB care, amongst HIV-negative patients.

## Methods

Here we give an outline of the model structure, inputs, and intervention scenarios, with further technical details given in S1 Text.

### Model structure

**Fig 1:**
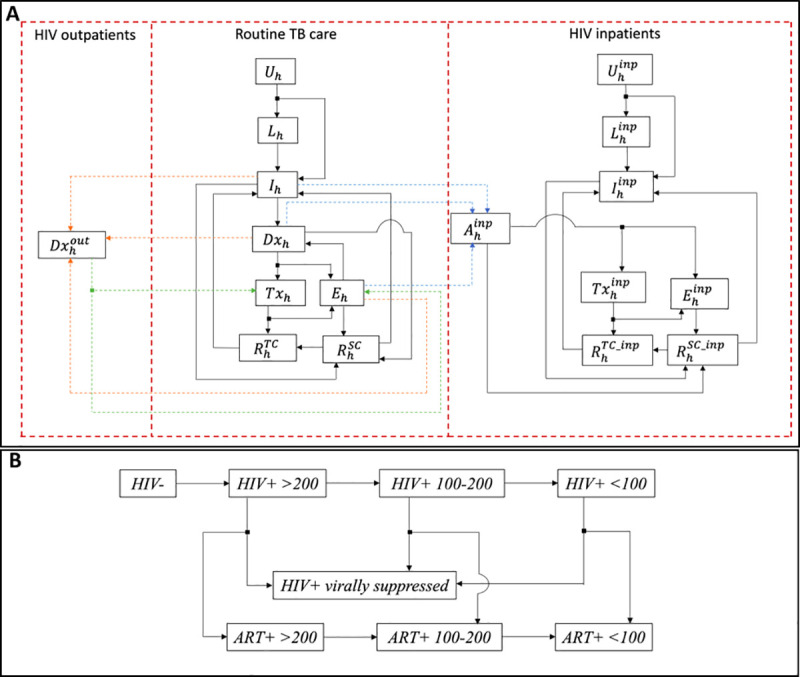
Schematic illustration of the model structure. (A) represents tuberculosis (TB) transmission dynamics in the general community (centre) and hospitalised patients living with HIV, represented by superscript ‘inp’ (right). Model compartments are as follows: uninfected with TB (*U*), latent infection (*L*), active TB disease (*I*), presented for care and awaiting diagnosis (*Dx*), on TB treatment (*Tx*), temporarily dropped out of the TB care cascade due to missed diagnosis or pre-treatment loss to follow-up (*E*), recovered from TB (*R*), and inpatient admissions (*A*). Each compartment is stratified further by HIV status, *h*, defined in Table B in S1 Text. (B) illustrates the transitions between the different HIV states, including antiretroviral therapy (ART) state and CD4 count level (cells/μl). The following states are also further stratified into pulmonary TB and extrapulmonary TB: *I*, *Dx*, *Tx*, *E*, *R*^TC^, *R*^SC^_,_ *Dx*^out^, *I*^inp^, *A*^inp^, *Tx*^inp^, *E*^inp^, *R*^TC_inp^, and *R*^SC_inp^. Arrows represent transitions between states, at the per capita rates listed in Table 2. Coloured-dash arrows in (A) illustrate movement of individuals with active TB disease into outpatient status (orange), out of outpatient status (green), and into inpatient status (blue). See S1 Text for further technical details, including model equations and calibration.

We developed a deterministic compartmental model of TB transmission amongst adults (>15 years old) in South Africa, incorporating the role of HIV in driving TB dynamics. The overall model structure is illustrated schematically in Fig 1. We did not aim to model the dynamics of HIV separately as we were interested in the effect of LAM tests on the TB epidemic, and not the HIV epidemic; thus, for the purpose of this analysis, we took the incidence of HIV, the proportion of people living with HIV (PLHIV) with and without TB, their CD4 cell counts, and the coverage of antiretroviral therapy (ART) over time as given inputs for the model. Doing so allows us to capture the role of projected future ART coverage, and HIV trends, in the future trajectory of the TB epidemic in South Africa. We also developed a similar model of TB transmission in Kenya, a country with a lower HIV burden.

**Table 2:** List of model parameters and assumptions.

Parameter	Symbol	Value (95% credible interval)		Source/notes
**TB natural history**				
Mean rate of transmission per TB case	β	14.9 (11.4–18.6)		Model estimate
TB infectiousness in HIV+ (not virally suppressed) relative to HIV−	κ_1_	0.66 (0.60–0.80)		Bacaër (2008) [16] Cohen (2006) [17]
Proportion of TB infections undergoing rapid progression	θ_h_	HIV− and virally suppressed	0.14 (0.115–0.16)	Vynnycky (1997) [18] Dye (1998) [19] Daley (1992) [20]
		HIV+, CD4 > 200	0.40 (0.33–0.51)	
		HIV+, CD4 100–200	0.80 (0.67–0.82)	
		HIV+, CD4 < 200	0.99 (0.90–1.00)	
Per capita hazard of reactivation of latent TB infection	ρ_h_	HIV− and virally suppressed	0.001 years^−1^ (0.0003–0.0024)	Horsburgh (2010) [21] Dye (1998) [19] Badri (2002) [22]
		HIV+, CD4 > 200	0.005 years^−1^ (0.003–0.006)	
		HIV+, CD4 100–200	0.10 years^−1^ (0.085–0.150)	
		HIV+, CD4 < 200	0.20 years^−1^ (0.100–0.300)	
Per capita hazard rate of relapse	*r*_1_	Following self-cure	0.06 years^−1^ (0.05–0.08)	Marx (2012) [23] Driver (2001) [24] Menzies (2009) [25] Thomas (2005) [26]
	*r*_2_	Following treatment cure	0.002 years^−1^ (0.001–0.003)	
Stabilisation of relapse risk following treatment	ς	0.5 years^−1^ (0.4–0.6)		Thomas (2005) [26], and assuming that relapse risk is highest in the first 2 years after recovery
Per capita hazard rate of spontaneous cure	φ_h_	HIV− and virally suppressed	0.17 years^−1^ (0.15–0.25)	Tiemersma (2011) [27]
		HIV+	0 years^−1^	
Per capita hazard rate of TB mortality	μ_2_		0.37 years^−1^ (0.31–0.59)	Model estimate
	μ_4_	Excess: inpatient	1 year^−1^ (0.5–2.0)	8-week mortality corresponds to Gupta-Wright (2018) [10]
Reduction in susceptibility due to previous infection	π	0.79 (0.25–0.85)		Andrews (2012) [28]
Proportion of TB cases with pulmonary TB	α_h_	HIV−	0.89 (0.80–0.90)	WHO (2019) [2]
		HIV+	0.60 (0.50–0.70)	
**HIV natural history**				
Per capita hazard rate of HIV acquisition	γ	0.016 years^−1^ (0.013–0.021)		Model estimate, to match HIV incidence
Per capita hazard rate of CD4 progression, amongst those not virally suppressed	η_1_	>200 to 100–200	0.31 years^−1^ (0.26–0.37)	Model estimate
	η_2_	100–200 to <100	0.89 years^−1^ (0.88–0.90)	
Per capita hazard rate of HIV mortality	$\mu_{3_{h}}$	HIV+ and virally suppressed	0.17 years^−1^ (0.15–0.19)	Model estimate
		Excess: HIV+, CD4 > 200	0.04 years^−1^ (0.02–0.06)	Anglaret (2012) [29] Badri (2006) [30] Mangal (2017) [31]
		Excess: HIV+, CD4 100–200	0.17 years^−1^ (0.13–0.21)	
		Excess: HIV+, CD4 < 100	0.60 years^−1^ (0.42–0.83)	
**Routine TB services**				
Per capita rate of initial careseeking for TB symptoms	δ_1_	HIV− and virally suppressed	2.5 years^−1^ (2.2–2.9)	Model estimate
Per capita rate of repeat careseeking for TB symptoms (following missed diagnosis)	δ_2_	HIV− and virally suppressed	12 years^−1^ (6–24)	Assumption: corresponds to range of 2 weeks to 2 months
Rate of careseeking amongst HIV+, relative to HIV−	κ_2_	1.5 (1.0–2.0)		Model estimate
Proportion of TB cases diagnosed correctly, per careseeking attempt in routine TB services	ε_R_	HIV− pulmonary TB	0.73 (0.63–0.92)	Model estimate
	κ_3_	HIV+, relative to HIV−	0.31 (0.20–0.72)	
	κ_4_	Extrapulmonary, relative to pulmonary	0.21 (0.20–0.28)	
TB treatment initiation delay	ϕ_R_	52 years^−1^ (24–100)		Assumed: corresponds to a mean treatment delay of 1 week in routine TB care
Proportion of diagnosed TB cases successfully initiating treatment	ω_R_	0.88 (0.77–0.90)		Naidoo (2017) [32] Maraba (2018) [33]
Rate of first-line treatment completion	τ	2 years^−1^		Corresponds to a duration of 6 months
Proportion cured after first-line treatment	χ_h_	HIV−	0.82 (0.77–0.87)	WHO (2019) [2] Naidoo (2017) [32]
		HIV+	0.80 (0.75–0.85)	
Proportion that can provide a sputum sample	$\vartheta_{h}^{sputum}$	HIV−	0.90 (0.80–0.95)	Geldenhuys (2014) [34] Lawn (2015) [35] Boyles (2018) [36] Gupta-Wright (2018) [10]
		HIV+	0.50 (0.37–0.60)	
Proportion that can provide a urine sample	$\vartheta_{h}^{urine}$	0.99 (0.97–1.00)		Broger (2019) [9]
Proportion of Xpert-negative results that are clinically diagnosed	ι_h_	HIV−	0.20 (0.16–0.24)	Naidoo (2017) [32]
		HIV+	0.30 (0.24–0.36)	
**HIV services**				
Per capita rate of hospitalisation	υ_h_	HIV+, CD4 > 200	0.046 years^−1^ (0.032–0.063)	Model estimate
		HIV+, CD4 100–200	0.12 years^−1^ (0.08–0.14)	
		HIV+, CD4 < 100	0.23 years^−1^ (0.20–0.26)	
Per capita rate of ART initiation in outpatient settings amongst PLHIV	ϖ_h_	HIV+, CD4 > 200	0.043 years^−1^ (0.020–0.085)	Model estimate
		HIV+, CD4 100–200	0.15 years^−1^ (0.09–0.27)	
		HIV+, CD4 < 100	0.28 years^−1^ (0.18–0.43)	
Proportion on ART that are virally suppressed	ϱ	0.88 (0.85–0.90)		UNAIDS estimates [37]
Inpatients: Amongst admissions with TB, proportion having TB symptoms	s_I_	0.95 (0.76–1.00)		Kerkhoff (2017) [38]
Inpatients: Amongst admissions with TB, proportion receiving an Xpert test at baseline	ξ_I_	1.00 (0.80–1.00)		Assuming proportion amongst inpatients is higher than amongst outpatients (see outpatient data below)
Inpatients: Proportion of diagnosed TB cases successfully initiating treatment	ω_I_	1.00 (0.90–1.00)		Naidoo (2017) [32] Maraba (2018) [33]
Inpatients: Average duration of hospitalisation	ψ_h_	HIV+, CD4 > 200	8 days (5–10)	Meyer-Rath (2013) [39]
		HIV+, CD4 100–200	9 days (6–11)	
		HIV+, CD4 < 100	10 days (7–13)	
Outpatients: Amongst those initiating ART, proportion having TB symptoms	*s*_O_	0.82 (0.66–0.98)		Drain (2015) [40]
Outpatients: Amongst those initiating ART, proportion of symptomatic patients currently receiving an Xpert test	ξ_O_	0.80 (0.60–0.90)		Christian (2018) [41]
Outpatients: Proportion of diagnosed TB cases successfully initiating treatment	ω_O_	0.86 (0.70–0.92)		Naidoo (2017) [32] Maraba (2018) [33]
TB treatment initiation delay	ϕ	365 years^−1^ (52–365)		Corresponds to a mean delay of 1 day
Proportion of diagnosed TB cases successfully initiating treatment	ε_O_	Outpatients	0.86 (0.70–0.92)	Naidoo (2017) [32] Maraba (2018) [33]
	ε_I_	Inpatients	1.00 (0.90–1.00)	
**Demographics**				
Per capita hazard rate of background mortality	μ_1_	0.0156 years^−1^		World Bank estimates: corresponds to an average lifespan of 65 years

CD4 counts are cells/μl. For further technical details and model specification, see S1 Text.

ART, antiretroviral therapy; PLHIV, people living with HIV; TB, tuberculosis.

Patients with extrapulmonary TB are often misdiagnosed if tested with sputum-based diagnostics (Table 2). To capture the advantages of LAM testing for diagnosing TB amongst these patients, in the model we distinguished extrapulmonary and pulmonary TB, while assuming that only the latter contribute to transmission. The sensitivity of LAM tests depends on the extent of HIV infection, and in particular the CD4 cell count [12]. Accordingly, amongst those with HIV, we modelled 3 different CD4 cell count strata: those with a CD4 count > 200 cells/μl, those with a CD4 count between 100 and 200 cells/μl, and those with a CD4 count < 100 cells/μl. The model captures the rate at which those with HIV progress through declining CD4 counts, during the course of infection. We also incorporated HIV-associated hospitalisation, assuming CD4-dependent hazard rates of admission into hospital, and further assuming that upon admission, any ART-naïve patients are initiated on ART. We also captured the provision of HIV care in outpatient settings, assuming CD4-dependent rates of ART initiation in these settings. The model does not explicitly capture rifampicin-resistant or multi-drug-resistant TB, as these forms account only for 3%–4% of overall TB burden in South Africa.

### Data sources

We drew from WHO data for estimates of TB incidence and mortality in South Africa, along with reported notifications and treatment outcomes [2]. For past HIV trends, we drew estimates from UNAIDS [37] for annual HIV incidence, the proportion of HIV cases being initiated on ART each year, and the proportion of those on ART being virally suppressed. For future projections, we drew from the Thembisa model [42], an HIV modelling framework that is the source of UNAIDs estimates (S5 and S6 Figs). Table 3 summarises the data for South Africa from these sources.

**Table 3:** Calibration targets for South Africa used to estimate model parameters.

Indicator		Value (95% credible interval)	Source
*TB epidemiology*			
TB incidence, 2018		520 per 100,000 (373–691)	WHO (2019) [2]
Mortality, 2018	HIV−	37 per 100,000 (35–39)	
	HIV+	73 per 100,000 (51–99)	
Notification rate, 2018		420 per 100,000 (358–438)	
*HIV epidemiology and care*			
HIV prevalence, 2017		7.5 million (6.9 million–8.0 million)	AIDSinfo [37]
Proportion of TB cases coinfected with HIV		0.59 (0.55–0.65)	WHO (2019) [2]
Proportion of PLHIV who have suppressed viral load		0.64 (0.58–0.68)	AIDSinfo [37]
Of ART-naïve patients in the community, proportion by CD4 category	CD4 > 200	0.76 (0.61–0.91)	Kharsany (2018) [43], Boyer (2016) [44], Huerga (2018) [45]
	CD4 100–200	0.12 (0.10–0.14)	
	CD4 < 100	0.12 (0.10–0.14)	
Of people initiating ART in outpatient settings, proportion by CD4 category	CD4 > 200	0.42 (0.33–0.49)	d’Elia (2015) [46], Drain (2017) [47], Brennan (2018) [48], Larsen (2019) [49], Bock (2018) [50], Cholera (2017) [51], Budgell (2015) [52]
	CD4 100–200	0.26 (0.20–0.30)	
	CD4 < 100	0.32 (0.24–0.36)	
Of people initiating ART as inpatients, proportion by CD4 category	CD4 > 200	0.34 (0.27–0.41)	Kerkhoff (2017) [38], Long (2016) [53], Broger (2019) [9]
	CD4 100–200	0.25 (0.20–0.28)	
	CD4 < 100	0.41 (0.33–0.45)	
Overall number initiating ART, 2017		660,982	AIDSinfo [37]
Percentage of HIV cases being hospitalised annually		5% (3.5%–6.5%)	Meyer-Rath (2013) [39]

CD4 counts are cells/μl.

ART, antiretroviral therapy; PLHIV, people living with HIV; TB, tuberculosis.

We performed a literature search to identify the proportions of PLHIV in the 3 different CD4 strata described above, stratifying by 3 different population types, in line with WHO recommendations: (i) those initiating ART upon admission to hospital, (ii) those initiating ART as outpatients, and (iii) those who are not on ART. S2 Fig summarises the proportions thus extracted, and the sources of data used. These data inform model estimates for the timeliness of HIV treatment as follows: Early treatment yields a patient population with higher CD4 counts at the point of treatment initiation, and, conversely, late treatment is associated with a patient population having lower CD4 counts (Table 3; S2 Fig). Therefore, by fitting the model to simultaneously capture CD4 progression and the distribution of CD4 counts at treatment initiation, we estimated the rate of treatment initiation at different CD4 counts, both in outpatient and inpatient settings (Table 3; S2 Fig). We additionally collected data from the literature for other parameters: for example, for the proportion of HIV cases that are hospitalised per year [39] and for the current standard of TB care amongst HIV inpatients (e.g., the proportion of hospital admissions receiving a TB test in routine practice) [10].

Using adaptive Markov chain Monte Carlo (MCMC), in particular the adaptive algorithm first proposed by Haario et al. [54], we incorporated uncertainty in model inputs (Table 2), propagating this input uncertainty into uncertainty in model projections. We drew 5,000 samples from the posterior distribution. For any model projections based on these samples, we estimated uncertainty intervals using the 2.5th and 97.5th percentiles, referring to this estimate as the Bayesian credible interval (CrI). Further details on the model structure and calibration are given in S1 Text.

### Intervention scenarios

We distinguished ‘current’ and potential ‘future’ LAM tests. For the performance of the former, we drew from a systematic review of LF-LAM [12]. For the latter, we took SILVAMP-LAM as an illustrative example, drawing from a recent study that estimated the performance of this test in HIV-negative patients [14]. This same study highlighted the potential of future LAM tests to have improved performance compared to SILVAMP-LAM: Our parameters for future LAM tests could therefore be interpreted as a lower bound for their performance [9] (Table 4). We assumed that differences in test performance between inpatients and outpatients are driven primarily by variations in CD4 distributions between these populations, variations that are captured by the model. Accordingly, we concentrated on study findings stratified by CD4 status, rather than by inpatient or outpatient setting.

**Table 4:** Test sensitivities.

Test	Symbol	HIV−	HIV+				Source
			CD4 > 200	CD4 100–200	CD4 < 100	Virally suppressed	
Sputum Xpert	*X*_sn_	89% (85–92)	79% (70–86)	79% (70–86)	79% (70–86)	79% (70–86)	Steingart (2014) [4]
Currently licensed LAM test	*A*_sn_	—	12% (5–24)	26% (15–39)	57% (42–70)	42%^a^ (32–52)	Bjerrum (2019) [12] Broger (2019) [9]
Future LAM test (consistent with SILVAMP-LAM as illustrative example)	*F*_sn_	30% (20–50)	44% (30–59)	61% (44–73)	84% (71–91)	70%^a^ (53–83)	Broger (2019) [9] Broger (2020) [14]

CD4 counts are cells/μl. Values in parentheses are 95% credible intervals. We assumed Xpert sensitivity to be independent of CD4 count amongst those with HIV.

^a^Sensitivities for patients with virally suppressed HIV infection were estimated by taking the ‘overall’ sensitivities of each test presented in the meta-analysis by Bjerrum et al. [12].

LAM, lipoarabinomannan.

A test’s epidemiological impact (i.e., on incidence and mortality) is driven by its sensitivity, or the proportion of true TB cases it can detect. A test’s specificity, or the proportion of those without TB that it correctly diagnoses as negative, has no direct bearing on its epidemiological impact, and instead is more relevant for the number of unnecessary TB treatments incurred as a result of false-positive diagnosis [55]. As the focus of the current work is on epidemiological impact, we concentrated on test sensitivity and not specificity. Our model therefore does not address the adverse consequences of false-positive results, including additional costs and adverse treatment side effects.

We modelled the deployment of current and future LAM tests in 2 intervention scenarios reflecting models of use in the updated WHO guidelines [13] (Table 1). In ‘PLHIV inpatients only’ (scenario i), LAM testing is conducted in PLHIV inpatients with signs and symptoms of TB and in all PLHIV inpatients with CD4 < 200 cells/μl, independent of symptoms. In ‘PLHIV inpatients and outpatients’ (scenario ii), LAM testing is conducted in PLHIV inpatients and outpatients prior to initiating ART treatment and with signs and symptoms of TB, in all PLHIV inpatients with CD4 < 200 cells/μl, and in all PLHIV outpatients with CD4 < 100 cells/μl, independent of symptoms. Additionally, for future LAM tests alone, we modelled a hypothetical future scenario: ‘universal for all TB presumptive patients’ (scenario iii), in which a future LAM test is deployed as part of routine TB diagnosis in patients presenting with symptoms of TB to a healthcare provider, regardless of HIV status. The impact of this scenario derives from the diagnosis of HIV-negative TB, and from the diagnosis of TB amongst those with undiagnosed HIV (and who may miss the opportunity for urine-based testing under current LAM testing guidelines). To assess which of these factors are most influential for impact, we additionally simulated scenario iii under a hypothetical condition where SILVAMP-LAM has 0 sensitivity for TB in HIV-negative individuals. This artificial scenario is thus deliberately constructed so that the only incremental cases being diagnosed, relative to scenario ii, are those with HIV and not on ART.

We modelled each of these intervention scenarios, assuming them to be initiated in 2020; we assumed that the proportion of the target population accessing these tests increases linearly, until the whole population in South Africa is covered by 2023. We simulated the model forward to 2035, simulating incidence and mortality over this time.

We considered the impact of LAM testing with respect to 2 comparators: (i) a ‘status quo’ comparator scenario, where the current standard of TB care continues indefinitely (where a proportion of patients presenting to care with signs and symptoms of TB are offered an Xpert test by a healthcare provider) and (ii) an ‘Xpert scale-up’ comparator scenario, involving the scale-up of sputum-based Xpert across the country, to diagnose TB both amongst those receiving ART and in routine TB services. We assumed that all patients presumed to have TB based on symptoms receive Xpert testing by 2023 (in inpatient, outpatient, and routine care settings).

Consistent with diagnostic yields reported in the literature (Table 2), we assumed that only 50% of patients with HIV (in both inpatient and outpatient settings) are able to provide a sputum sample for Xpert testing, while 99% of patients are able to provide a urine sample for LAM testing. We also considered clinical diagnosis that occurs after a negative Xpert test result. We assumed that 20% and 30% of patients with negative Xpert test results are clinically diagnosed and offered treatment amongst HIV-negative and HIV-positive patients, respectively [32]. Using comparator and intervention scenarios, we projected estimates for the numbers of TB cases and deaths that would be averted under the intervention scenarios described above.

Finally, as a sensitivity analysis to examine the applicability of our results to other countries with a generalised HIV epidemic, we extended our model to capture epidemiological conditions consistent with Kenya, where an estimated 27% of TB is in HIV-coinfected patients, compared with 59% for South Africa [2]. There is insufficient data from Kenya to calibrate all model parameters; we therefore sought only to capture gross epidemiological indicators consistent with Kenya (TB incidence, mortality, proportion of HIV coinfection, etc.), while assuming the same values as derived for South Africa for all parameters specific to CD4 counts (rates of ART initiation, hospitalisation, etc.; see S2 Table for further details). We then simulated the intervention scenarios described above, in this Kenya-like setting.

## Results

S1 Fig shows results of model calibration, displaying the model fit against each of the calibration targets listed in Table 3. Our model projects that, under the status quo comparator between 2020 and 2035, there would be 2,700,000 (95% CrI 2,000,000–3,600,000) cumulative incident cases of TB in South Africa, and 420,000 (95% CrI 350,000–520,000) cumulative TB deaths.

**Fig 2:**
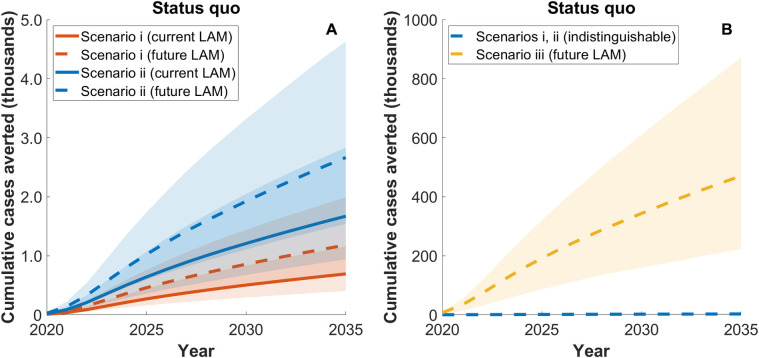
Model projections for impact of LAM tests in South Africa on TB incidence, relative to the status quo comparator. Under the status quo comparator, the current standard of TB care in South Africa is assumed to continue indefinitely. Shaded areas show Bayesian 95% credible intervals. Solid lines depict a currently licensed test, while dashed lines depict a future LAM test. Colours represent different implementation scenarios: inpatients only (red, scenario i), plus outpatients (blue, scenario ii) and plus routine TB care (yellow, scenario iii). (A) depicts scenarios i and ii only, while (B) additionally shows scenario iii (shown separately owing to the change in scale). Cumulative impacts over the period 2020–2035 are summarised in Table 5. LAM, lipoarabinomannan; TB, tuberculosis.

**Fig 3:**
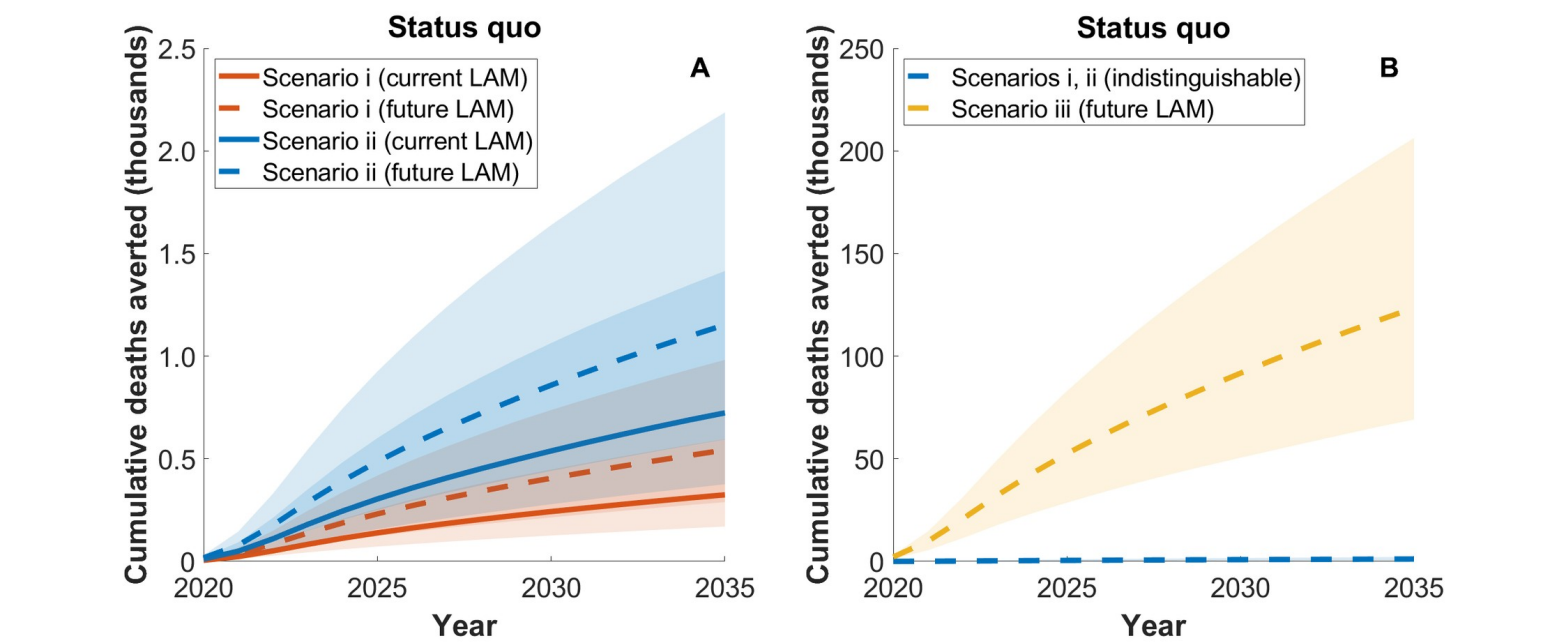
Model projections for impact of LAM test in South Africa on TB deaths, relative to the status quo comparator. Under the status quo comparator, the current standard of TB care in South Africa is assumed to continue indefinitely. Shaded areas show Bayesian 95% credible intervals. Solid lines depict a currently licensed test, while dashed lines depict a future LAM test. Colours represent different implementation scenarios: inpatients only (red, scenario i), plus outpatients (blue, scenario ii) and plus routine TB care (yellow, scenario iii). (A) depicts scenarios i and ii only, while (B) additionally shows scenario iii. Cumulative impacts over the period 2020–2035 are summarised in Table 5. LAM, lipoarabinomannan; TB, tuberculosis.

Fig 2 and Fig 3 illustrate the potential impact of LAM tests relative to the status quo comparator, showing the TB cases and deaths averted each year, respectively. Table 5 summarises the cumulative impact in South Africa over the period 2020 to 2035, showing estimates for both cases and deaths averted. As context to these results, S1 Table shows additional model outputs for the numbers of patients initiating HIV treatment in both outpatient and inpatient settings. Together these results illustrate that LAM tests could have a meaningful impact in saving lives amongst inpatients (scenario i); current and future LAM tests may avert, respectively, 54 (95% CrI 33–86) and 90 (95% CrI 55–145) TB deaths amongst inpatients, a 5.33% (95% CrI 4.18%–6.29%) and 8.75% (95% CrI 7.05%–11.2%) reduction of overall TB deaths in this population. At a population level, 324 (95% CrI 170–596) and 543 (95% CrI 289–982) TB deaths would be averted with current and future LAM tests, respectively, a limited impact (<1% of the country-level TB burden) that is clearly because of the small size of the population receiving the intervention.

**Table 5:** Projected cumulative impact relative to a status quo comparator, South Africa.

Deployment level	LAM test	Cumulative incidence averted, 2020–2035		Cumulative TB deaths averted, 2020–2035		Cumulative TB deaths averted amongst inpatients, 2020–2035	
		Number	Percent	Number	Percent	Number	Percent
Inpatients (scenario i)	Currently licensed LAM	692 (402–1,156)	0.026 (0.014–0.043)	324 (170–596)	0.077 (0.045–0.125)	54 (33–86)	5.33 (4.18–6.29)
	Future LAM	1,181 (698–1,990)	0.045 (0.025–0.074)	543 (289–982)	0.129 (0.076–0.203)	90 (55–145)	8.75 (7.05–11.2)
Inpatients and outpatients (scenario ii)	Currently licensed LAM	1,670 (937–2,837)	0.063 (0.035–0.109)	724 (376–1,415)	0.173 (0.097–0.306)	58 (36–93)	5.73 (4.36–6.90)
	Future LAM	2,665 (1,539–4,631)	0.100 (0.056–0.175)	1,153 (594–2,188)	0.276 (0.156–0.486)	96 (58–155)	9.33 (7.43–12.1)
Inpatients, outpatients, and routine TB care (scenario iii)	Future LAM	470,590 (221,830–871,500)	17.7 (8.62–29.0)	123,520 (69,120–206,270)	29.6 (17.8–43.6)	402 (229–685)	39.7 (29.4–49.1)

Under the status quo comparator, we assume the current standard of TB care in South Africa to continue indefinitely. Values in parentheses are 95% credible intervals.

LAM, lipoarabinomannan; TB, tuberculosis.

When expanding LAM test deployment to outpatient settings (scenario ii), current and future LAM tests would avert, respectively, 724 (95% CrI 376–1,400) and 1,200 (95% CrI 594–2,200) TB deaths, roughly doubling the total deaths averted under scenario i. Notably, however, even with this widened eligibility, LAM tests continue to exert only a modest impact on the population-level TB epidemic, with <1% reductions in TB incidence and mortality (Figs 2A and 3A; Table 5). It is only when LAM tests are deployed in routine TB services (scenario iii) that true incidence-reducing impact emerges (Figs 2B and 3B), with 470,000 (95% CrI 220,000–870,000) cumulative TB incident cases and 120,000 (95% CrI 69,000–210,000) TB deaths averted, respective reductions of 17.7% (95% CrI 8.62%–29.0%) and 29.6% (95% CrI 17.8%–43.6%). S7 Fig illustrates the reason for the limited population-level impact under scenarios i and ii, even in a high-HIV-burden setting such as South Africa: It is due to the series of criteria that successively narrow the pool of eligible individuals to <5% of annual TB incidence.

Table 6 summarises the cumulative impact under the Xpert scale-up comparator. In this case the incremental cases averted by a future LAM test in routine TB care (scenario iii) fall to 120,000 (95% CrI 69,000–170,000), a 5.68% (95% CrI 3.18%–7.52%) reduction from the 17.7% (95% Cri 8.62%–29.0%) achieved with the status quo comparator, owing to the improvements in diagnosis already achieved by Xpert scale-up. Under the same scenario, a future LAM test would avert 50,000 (95% CrI 34,000–73,000) TB deaths, a 16.4% (95% CrI 10.4%–22.2%) reduction. Overall, the effect of Xpert scale-up on the incremental impact of LAM tests is lower for deaths averted than for cases averted: This reflects the value of LAM tests in diagnosing extrapulmonary TB and TB in those with advanced disease, patients who would otherwise contribute more strongly to mortality than to transmission.

**Table 6:** Projected cumulative impact relative to an Xpert scale-up comparator, South Africa.

Deployment level	LAM test	Cumulative incidence averted, 2020–2035		Cumulative TB deaths averted, 2020–2035		Cumulative TB deaths averted amongst inpatients, 2020–2035	
		Number	Percent	Number	Percent	Number	Percent
Inpatients (scenario i)	Currently licensed LAM	171 (81–322)	0.0079 (0.0043–0.014)	153 (81–324)	0.050 (0.028–0.088)	34 (20–56)	4.68 (3.68–5.62)
	Future LAM	291 (136–546)	0.014 (0.0074–0.025)	256 (134–523)	0.083 (0.047–0.14)	56 (33–93)	7.69 (6.12–10.2)
Inpatients and outpatients (scenario ii)	Currently licensed LAM	395 (192–798)	0.019 (0.010–0.037)	330 (173–747)	0.108 (0.058–0.210)	36 (22–60)	4.99 (3.81–6.13)
	Future LAM	635 (293–1,327)	0.030 (0.016–0.060)	530 (271–1,143)	0.173 (0.094–0.329)	59 (35–98)	8.15 (6.41–10.8)
Inpatients, outpatients, and routine TB care (scenario iii)	Future LAM	119,670 (68,710–169,030)	5.68 (3.18–7.52)	50,174 (33,617–72,557)	16.4 (10.4–22.2)	204 (122–344)	28.7 (21.6–34.5)

Under the Xpert scale-up comparator, we assume comprehensive expansion of access to sputum Xpert across South Africa, such that all individuals with symptoms suggestive of TB are tested with Xpert on their first presentation for care. Numbers in the table show the model-projected impact of LAM tests when deployed as an adjunct to Xpert, assuming (in all settings shown in the table) a simple diagnostic algorithm where TB is diagnosed if either a LAM test or Xpert is positive for TB. Values in parentheses are 95% credible intervals. As described in the main text, in the model we also allow for clinical diagnosis amongst bacteriologically negative symptomatic patients.

LAM, lipoarabinomannan; TB, tuberculosis.

As noted above, the impact of scenario iii (Figs 2B and 3B) could derive either from diagnosis of TB in HIV-negative individuals or from diagnosis of TB amongst those with HIV who have not yet been linked to care. To examine the roles of these 2 populations, we simulated a hypothetical scenario of a future LAM test being deployed in routine TB services, but with 0 sensitivity for TB in HIV-negative individuals. Under this hypothetical scenario the TB cases and deaths averted are, respectively, 12.1% (95% CrI 8.34%–17.6%) and 21.3% (95% CrI 15.2%–29.7%) in South Africa, hypothetical impacts that represent only marginal reductions of those reported in the bottom row of Table 5. Overall, these findings illustrate that—when expanded from outpatients in HIV care to routine TB services—the key value of LAM tests would be in diagnosing TB amongst those with HIV who have not yet been initiated on ART.

Similar results are seen in a Kenya-like setting (S3 and S4 Tables; S9 Fig). Relative to a status quo comparator, in scenarios i and ii, where LAM tests are deployed only amongst those receiving HIV care, the percentage decline in mortality amongst eligible groups is roughly half that in South Africa (S3 Table), owing to the smaller proportion of HIV cases having TB. As in South Africa, population-level declines in incidence and mortality would be <1% in these scenarios. However, when deployment of a future LAM test is expanded to routine TB services, 290,000 (95% CrI 190,000–470,000) cumulative TB incident cases and 58,000 (95% CrI 39,000–86,000) TB deaths are averted, a 19.8% (95% CrI 18.1%–22.7%) and 27.9% (95% CrI 25.7%–31.4%) reduction, respectively, in a Kenya-like setting (S3 Table), impacts that are comparable to those estimated for South Africa (Table 5).

## Discussion

### Summary

New non-sputum-based diagnostic technologies could raise new opportunities to accelerate current declines in TB incidence [56–58]. In the current work we have addressed one example of such technologies: lateral flow LAM assays, which offer the potential to diagnose TB through urine. Given that LAM tests perform best in PLHIV [12], it was reasonable to hypothesise that they would have a substantial epidemiological impact in settings such as South Africa, where the majority of TB cases are HIV coinfected. However, our results suggest a more nuanced message: For future LAM tests to achieve population-level reductions in TB burden, they would need to be offered more widely than amongst those receiving HIV care, and in routine TB care. Under this scenario, the patients who would benefit are not just HIV-negative cases, but also those with HIV who have not yet been engaged in HIV care. The latter population could arise from a variety of factors, including the rate of HIV diagnosis or gaps in linkage to ART initiation after a diagnosis has been made. In practice, the feasibility of such wide deployment of LAM tests will depend critically on the performance characteristics of the tests concerned.

While much of the existing literature on LF-LAM addresses its diagnostic performance and implementation in defined clinical settings [9,10,12,14], our work complements this evidence basis by addressing the population level in countries with a high HIV and TB burden. To our knowledge, this is the first modelling study to examine the potential incidence and mortality impact that LF-LAM tests may offer.

### Limitations and key areas for future work

Although we expect similar results to apply in other settings with a generalised HIV epidemic, our analysis does not address high-TB-burden countries where HIV is not a driving factor, such as India. The potential use of LF-LAM in such settings is an important area for further work. In these settings, we expect that the value of a future LAM test might be driven not by its ability to detect TB amongst those with HIV, but rather by its potential to be used more widely than current diagnostic tools needing laboratory capacity: for example, in primary care and in peripheral healthcare settings.

Our work also does not address cost or cost-effectiveness, another important topic for future work. Recent analysis indicated that LF-LAM and SILVAMP-LAM would be cost-effective when deployed in inpatient settings in both Malawi and South Africa [59]; future work, especially in the context of improved-performance tests, could benefit from incorporating a transmission framework. Relatedly, we have also not addressed the staffing and health system capacity that would be needed to facilitate the expansion of LAM testing in South Africa [60], nor the costs associated with undertaking CD4 cell count measurements. It should be noted that CD4 cell count measurement is increasingly being replaced by viral load monitoring. In addition, we have not addressed potential implementation challenges in the use of future LAM tests. For example, SILVAMP-LAM involves an additional step compared to LF-LAM; findings from ongoing trials will be valuable in determining whether this procedure reduces the performance of this technology to any appreciable extent.

Amongst additional model simplifications, we have not covered paediatric TB, an important and underaddressed part of TB burden. We have also taken a country-level perspective, despite wide subnational variation in TB/HIV burden within South Africa [61,62]. For simplicity we have ignored drug resistance: Despite the potential benefits of LAM tests, one notable limitation of these tests is that they cannot determine drug sensitivity. In the model calibration, we have captured temporal trends and projections in major features of the HIV epidemic in South Africa, such as HIV incidence and the proportion on ART; however, we have modelled other features as static, for example the percentage of HIV cases that are hospitalised. Further data on how this proportion has changed over time should allow our model to better capture these dynamics, potentially affecting our estimates for deaths averted amongst those being hospitalised. However, we have shown that this impact accounts for only a small proportion of overall TB burden in South Africa; we therefore do not expect these changes to affect our overall qualitative findings on the importance of widened eligibility for epidemiological impact.

Given our focus on epidemiological impact, our analysis of test performance is limited to test sensitivity. However, for any future LAM test that is intended for use in routine TB services, test specificity will be a critical performance characteristic, in order to minimise the number of unnecessary TB treatments incurred [55]. As an illustrative example, a test having 95% specificity and 90% sensitivity would—for the 10% TB prevalence that is typical amongst symptomatic individuals presenting to routine TB services—result in 1 false-positive TB diagnosis for every 2 successful TB diagnoses, an unacceptably high rate. Evidence suggests that both LF-LAM and SILVAMP-LAM may have reduced specificity at lower CD4 counts [9,13]. Estimating this risk is an important area for future work: however, an important first step in this direction is to address the real uncertainties in quantifying specificity where the accuracy of the reference standard is unclear. An example of when a reference standard may be unclear includes the use of a sputum-based microbiological reference standard amongst patients with solely extrapulmonary TB, and potentially also among patients with HIV, who are more likely to produce paucibacillary sputum [6,63]. Although a LAM test may correctly diagnose such patients, this diagnosis would be deemed incorrect by a sputum-based microbiological reference standard. For future quantitative analysis, therefore, there is a need for more systematic estimates of specificity that take account of such shortcomings of any given reference standard. Combined use of microbiological and composite reference standards (i.e., including clinical diagnosis) may help in this regard.

## Conclusions

In this study, we observe that even in a setting with high HIV burden, such as South Africa, future LAM tests will need to have sufficient performance to be offered more widely than HIV care, in order to have incidence-reducing impact. Future LAM tests will also need to have sufficient specificity to achieve this impact without incurring an undue burden of unnecessary TB treatment. Any emerging LAM technology meeting these criteria would be invaluable in accelerating current declines in TB burden.

## Supporting information

S1 Fig Model fits to data for South Africa. Data points are described in Table 3. (A) TB incidence; (B) TB mortality per 100,000; (C) TB notifications per 100,000; (D) proportion of PLHIV with suppressed viral loads; (E) ART initiations per 100,000; (F) proportion of TB cases coinfected with HIV; (G) percentage of HIV cases being hospitalised annually.
(TIF)

S2 Fig CD4 count distributions. Model fits to the data: mean model CD4 count distributions amongst ART-naïve PLHIV (blue), inpatients (red), and outpatients (yellow). Grey lines show the model runs, and black data points show the data, based on a literature search (Table 3).
(TIF)

S3 Fig Markov chain Monte Carlo diagnostics. (A) shows the posterior density trace. (B) and (C) show autocorrelation function plots for 2 selected parameters (rate of transmission and proportion of fast progressors).
(TIF)

S4 Fig Multivariate sensitivity analysis of impact for South Africa. Using scenario iii (future LAM test deployment in routine TB care), we used the partial rank correlation coefficient (PRCC) to examine which parameter listed in Table 2 the output cases averted is most sensitive towards. Larger bars represent more sensitive parameters. Shown are the 20 most influential model parameters, in decreasing order of sensitivity from top to bottom.
(TIF)

S5 Fig Total new HIV infections in South Africa. Past estimates (in blue shading) and future projections (in green shading). (TIF)

S6 Fig Proportion of PLHIV on ART in South Africa. Past estimates (in blue shading) and future projections (in green shading).
(TIF)

S7 Fig Cumulative effect of eligibility criteria. Flow diagram illustrating the effect of the most recent eligibility criteria in WHO recommendations for the use of LF-LAM on the size of the subset of TB incident cases who receive LAM testing. In South Africa, of patients with TB disease, around 59% are coinfected with HIV; of these, 90% are aware of their HIV status, of which, approximately 18% will initiate ART per year. Depending on the eligibility criteria, around 10%–30% of individuals who initiate ART will be eligible for LAM testing. For the purpose of this illustration, of the 7,700,000 who are HIV-positive, 2,900,000 were estimated not to be on ART in 2018. Approximately 90% of these (2,610,000) are aware of their HIV status. Model estimates suggest that approximately 480,000 initiated ART in 2018. Thus, we approximate that in 2018, 18% of individuals who were aware of their HIV infection initiated ART (480,000/2,610,000).
(TIF)

S8 Fig Model fits to data for Kenya. Data points are described in S2 Table. (A) TB incidence; (B) TB mortality per 100,000; (C) TB notifications per 100,000; (D) proportion of PLHIV with suppressed viral loads; (E) ART initiations per 100,000; (F) proportion of TB cases coinfected with HIV; (G) percentage of HIV cases being hospitalised annually.
(TIF)

S9 Fig Model projections for impact of LAM tests in Kenya on TB incidence, relative to the status quo comparator. Under this comparator, the current standard of TB care in Kenya is assumed to continue indefinitely. Shaded areas show Bayesian 95% credible intervals. Solid lines depict a currently licensed test, while dashed lines depict a future LAM test. Colours represent different implementation scenarios: inpatients only (red, scenario i), plus outpatients (blue, scenario ii), and plus routine TB care (yellow, scenario iii). (A) depicts scenarios i and ii only, while (B) additionally shows scenario iii (shown separately owing to the change in scale). Cumulative impacts over the period 2020–2035 are summarised in S3 Table.
(TIF)

S1 Table. TB incidence and mortality, and number of patients initiating ART per year, in inpatient and outpatient settings (DOCX)

S2 Table Calibration targets for Kenya used to estimate model parameters. (DOCX)

S3 Table Projected cumulative impact relative to a status quo comparator, Kenya. (DOCX)

S4 Table Projected cumulative impact relative to an Xpert scale-up comparator, Kenya. (DOCX)

S1 Text Supplementary materials and methods. (DOCX)

## Abbreviations

ART: antiretroviral therapy
CrI: credible interval
LAM: lipoarabinomannan
PLHIV: people living with HIV
TB: tuberculosis
