# Supplementary figures and images for: The potential impact of urine-LAM diagnostics on tuberculosis incidence and mortality: A modelling analysis
Source: PLoS Med. 2020 Dec 11;17(12):e1003466. doi: 10.1371/journal.pmed.1003466 (PMC7732057; doi:10.1371/journal.pmed.1003466)

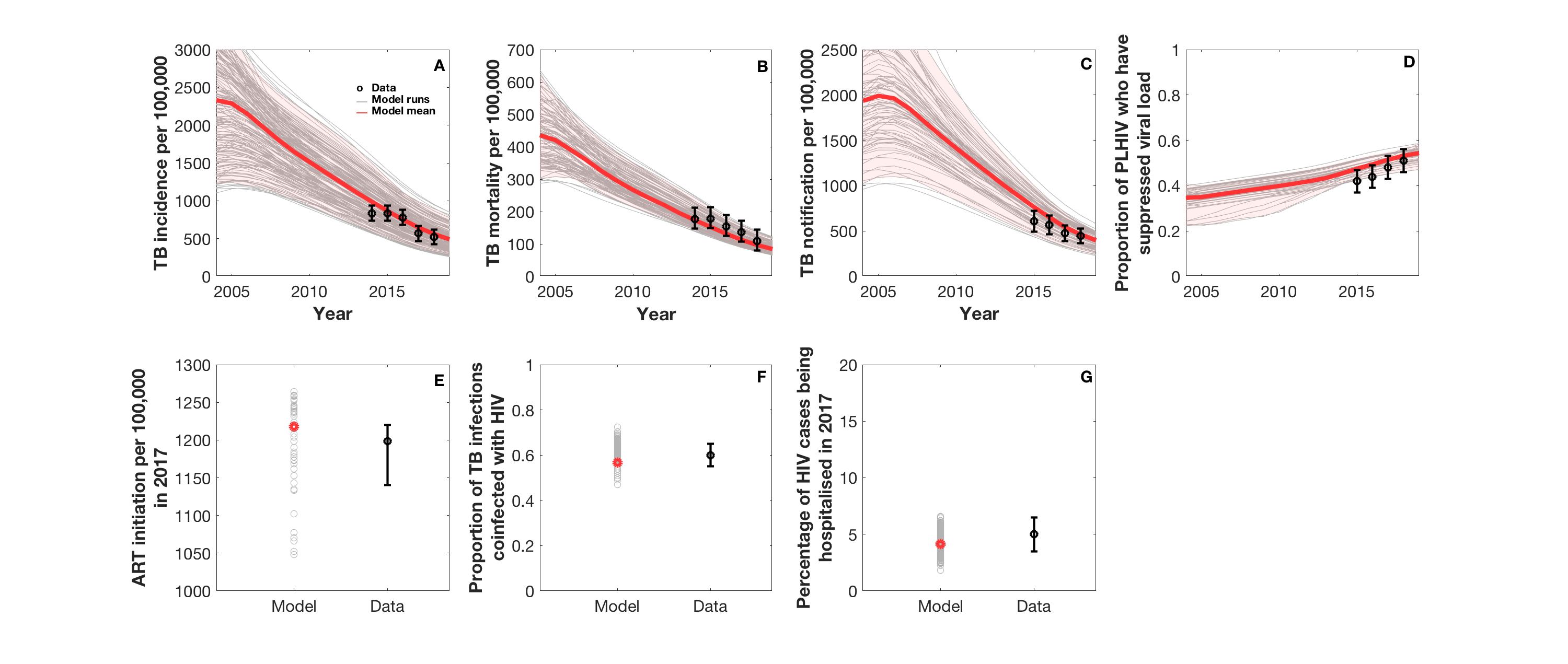

Supplement: S1 Fig — Data points are described in Table 3. (A) TB incidence; (B) TB mortality per 100,000; (C) TB notifications per 100,000; (D) proportion of PLHIV with suppressed viral loads; (E) ART initiations per 100,000; (F) proportion of TB cases coinfected with HIV; (G) percentage of HIV cases being hospitalised annually. (TIF) [file pmed.1003466.s001.tif]

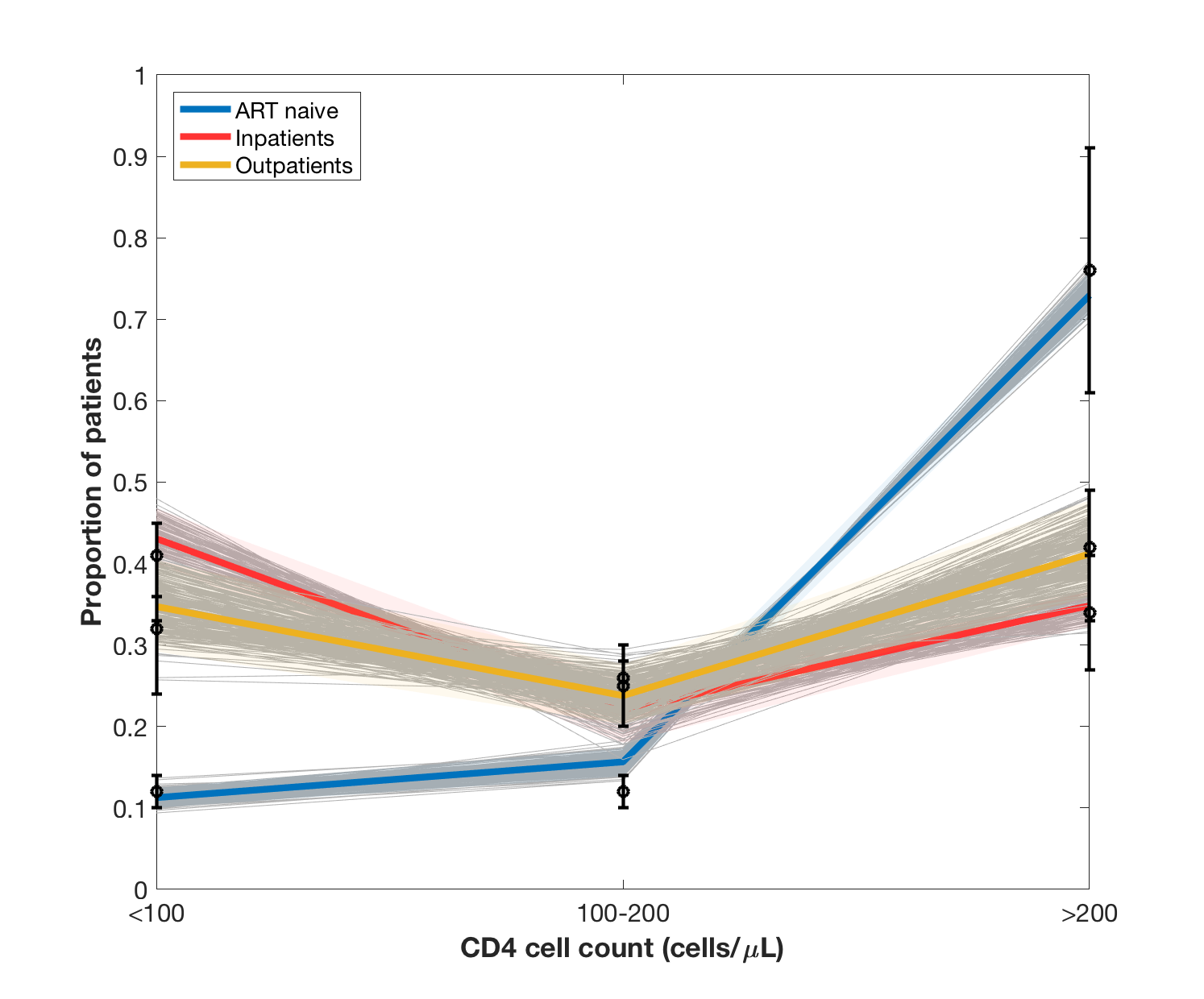

Supplement: S2 Fig — Model fits to the data: mean model CD4 count distributions amongst ART-naïve PLHIV (blue), inpatients (red), and outpatients (yellow). Grey lines show the model runs, and black data points show the data, based on a literature search (Table 3). (TIF) [file pmed.1003466.s002.tif]

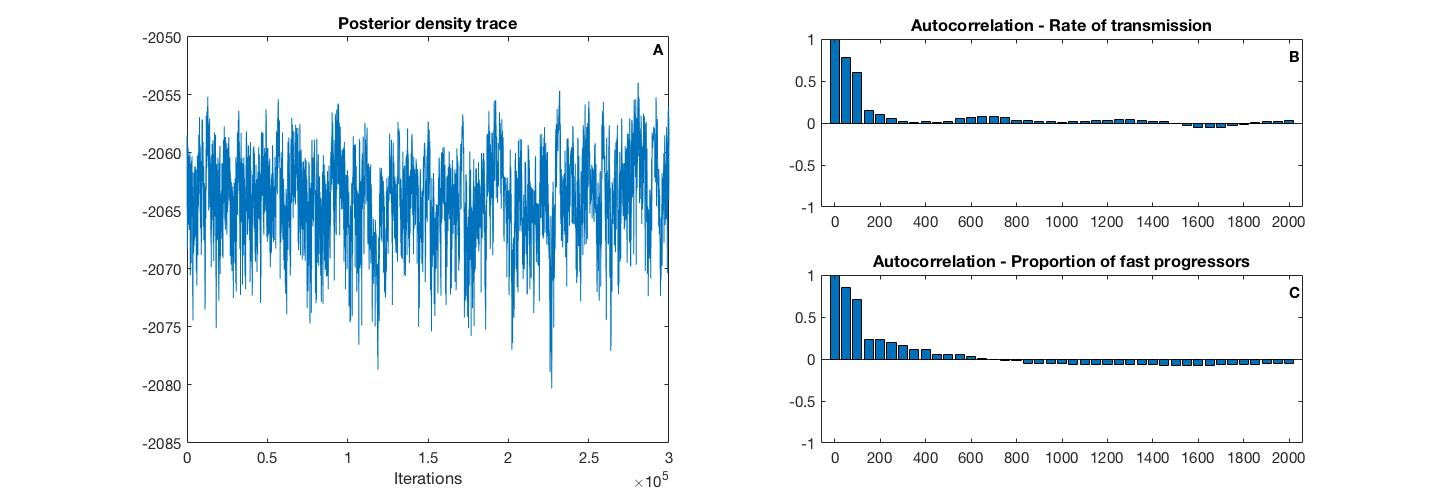

Supplement: S3 Fig — (A) shows the posterior density trace. (B) and (C) show autocorrelation function plots for 2 selected parameters (rate of transmission and proportion of fast progressors). (TIF) [file pmed.1003466.s003.tif]

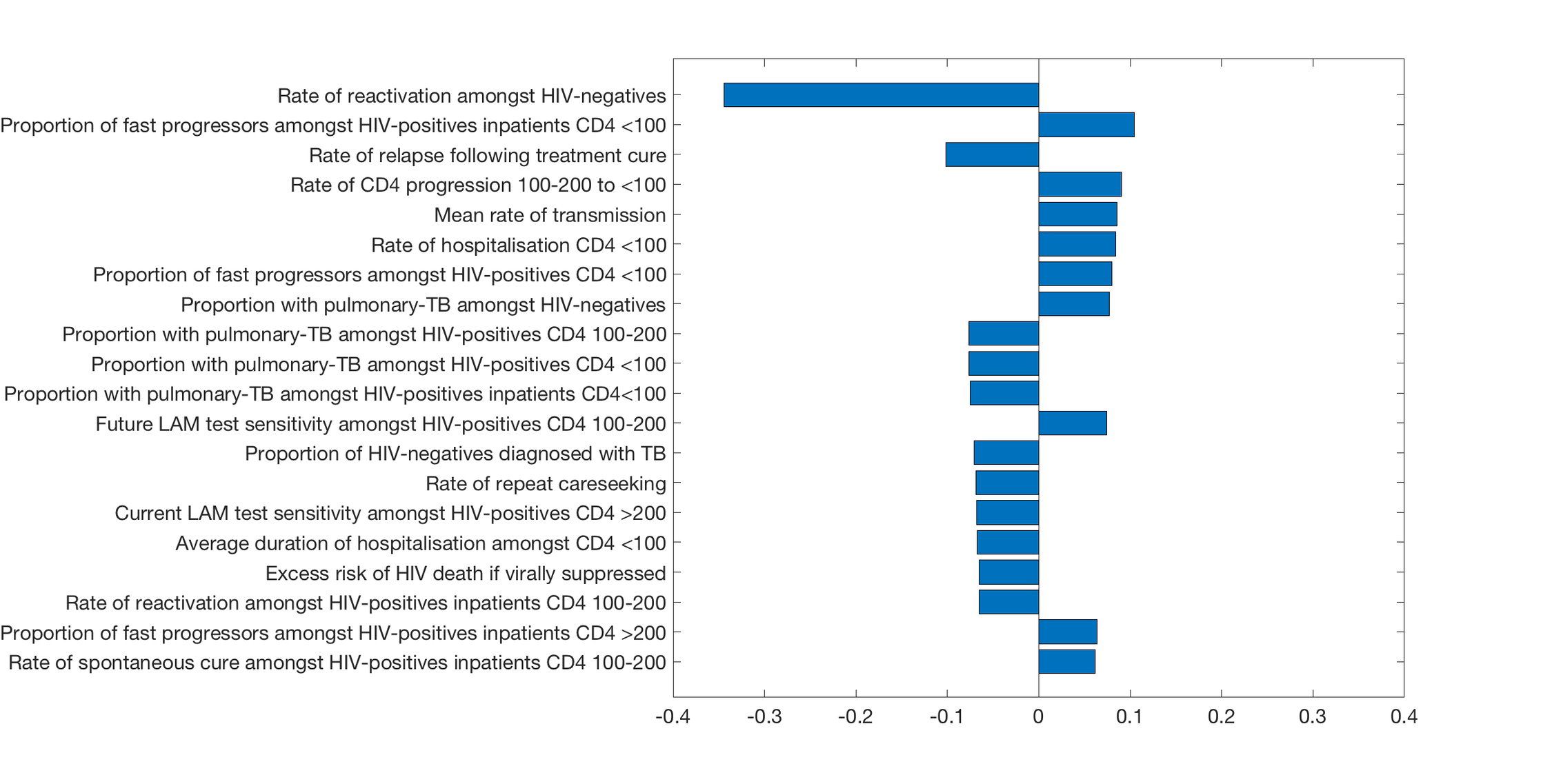

Supplement: S4 Fig — Using scenario iii (future LAM test deployment in routine TB care), we used the partial rank correlation coefficient (PRCC) to examine which parameter listed in Table 2 the output cases averted is most sensitive towards. Larger bars represent more sensitive parameters. Shown are the 20 most influential model parameters, in decreasing order of sensitivity from top to bottom. (TIF) [file pmed.1003466.s004.tif]

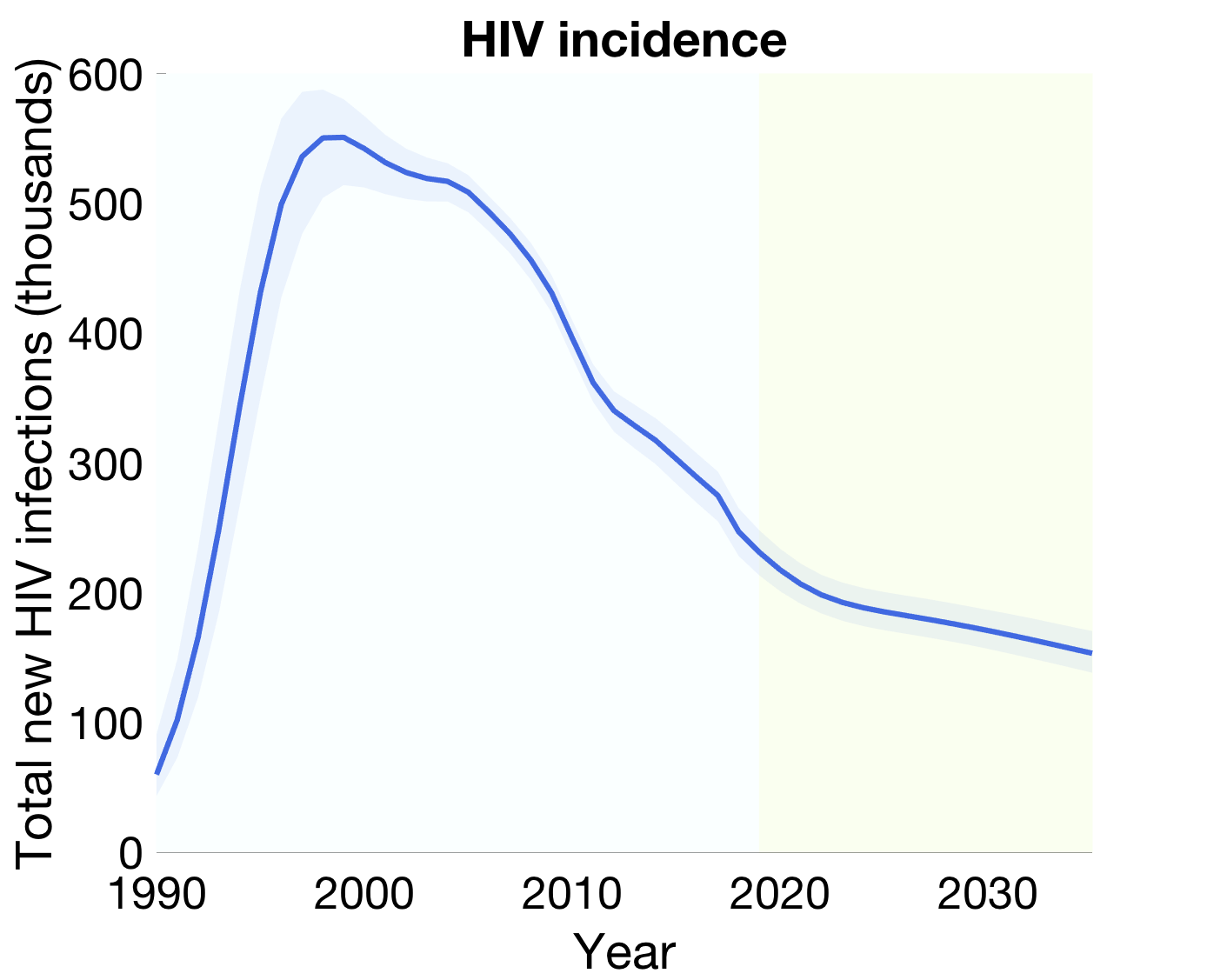

Supplement: S5 Fig — (TIF) [file pmed.1003466.s005.tif]

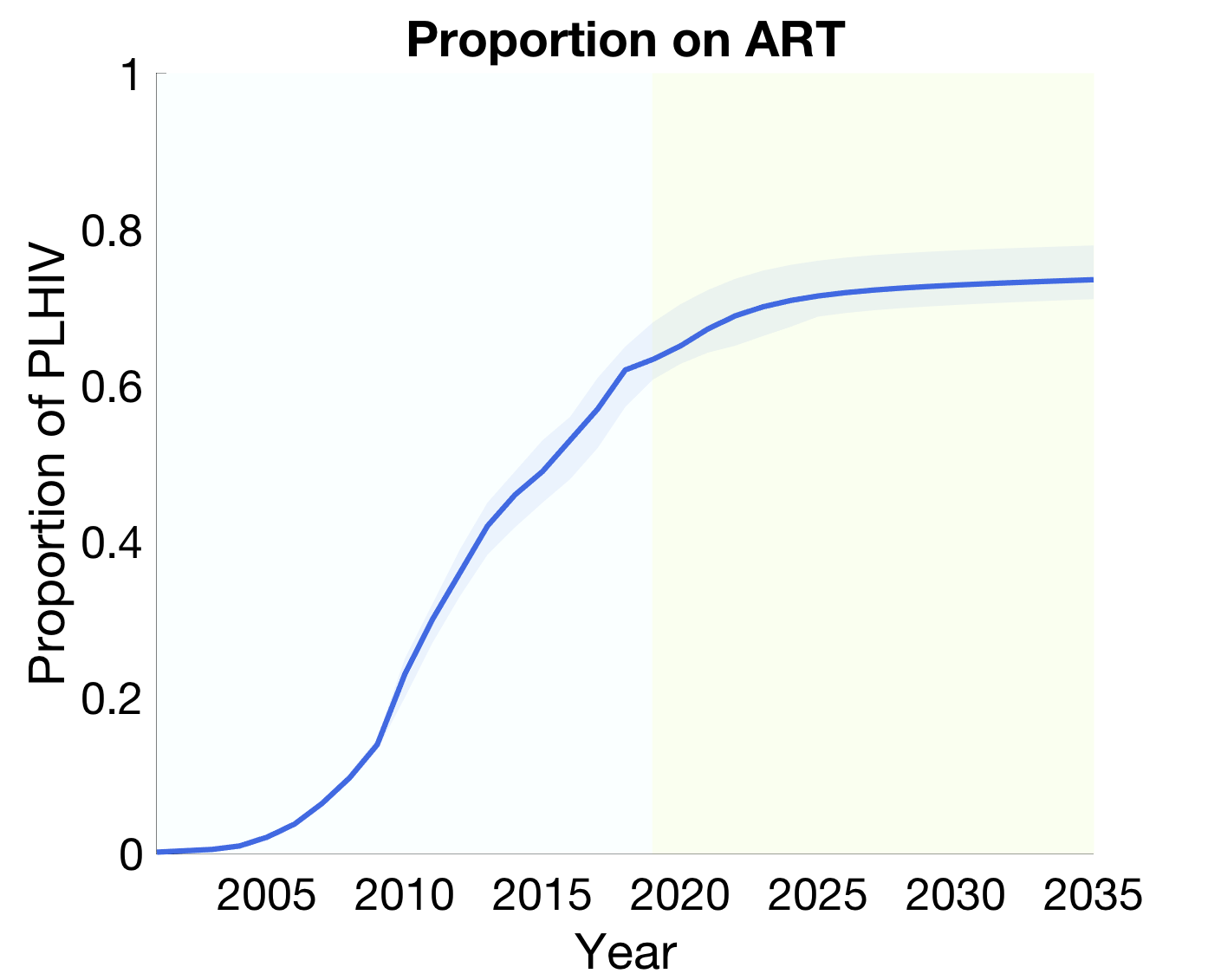

Supplement: S6 Fig — Past estimates (in blue shading) and future projections (in green shading). (TIF) [file pmed.1003466.s006.tif]

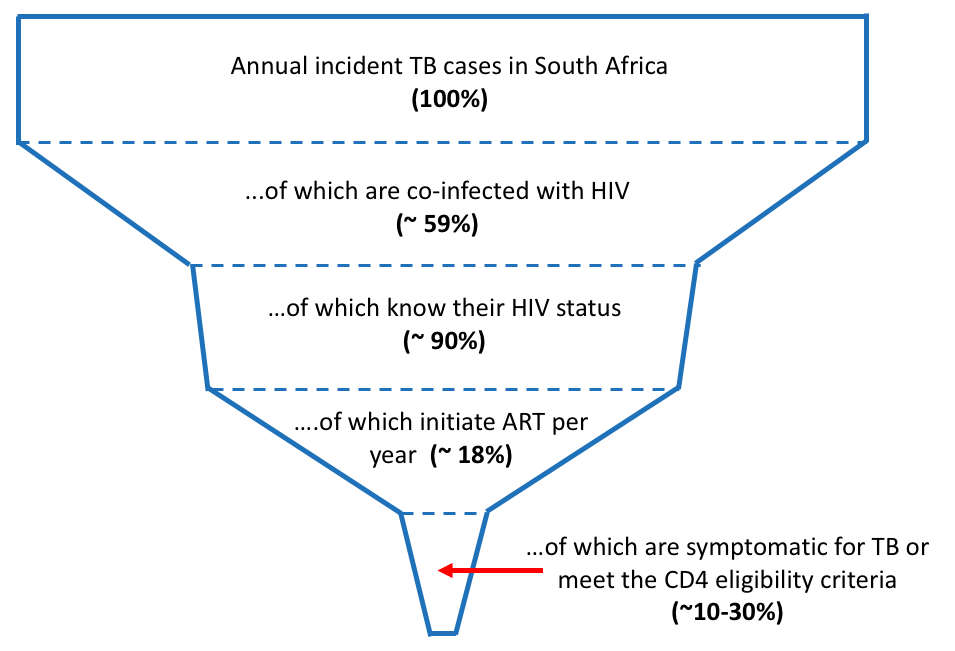

Supplement: S7 Fig — Flow diagram illustrating the effect of the most recent eligibility criteria in WHO recommendations for the use of LF-LAM on the size of the subset of TB incident cases who receive LAM testing. In South Africa, of patients with TB disease, around 59% are coinfected with HIV; of these, 90% are aware of their HIV status, of which, approximately 18% will initiate ART per year. Depending on the eligibility criteria, around 10%–30% of individuals who initiate ART will be eligible for LAM testing. For the purpose of this illustration, of the 7,700,000 who are HIV-positive, 2,900,000 were estimated not to be on ART in 2018. Approximately 90% of these (2,610,000) are aware of their HIV status. Model estimates suggest that approximately 480,000 initiated ART in 2018. Thus, we approximate that in 2018, 18% of individuals who were aware of their HIV infection initiated ART (480,000/2,610,000). (TIF) [file pmed.1003466.s007.tif]

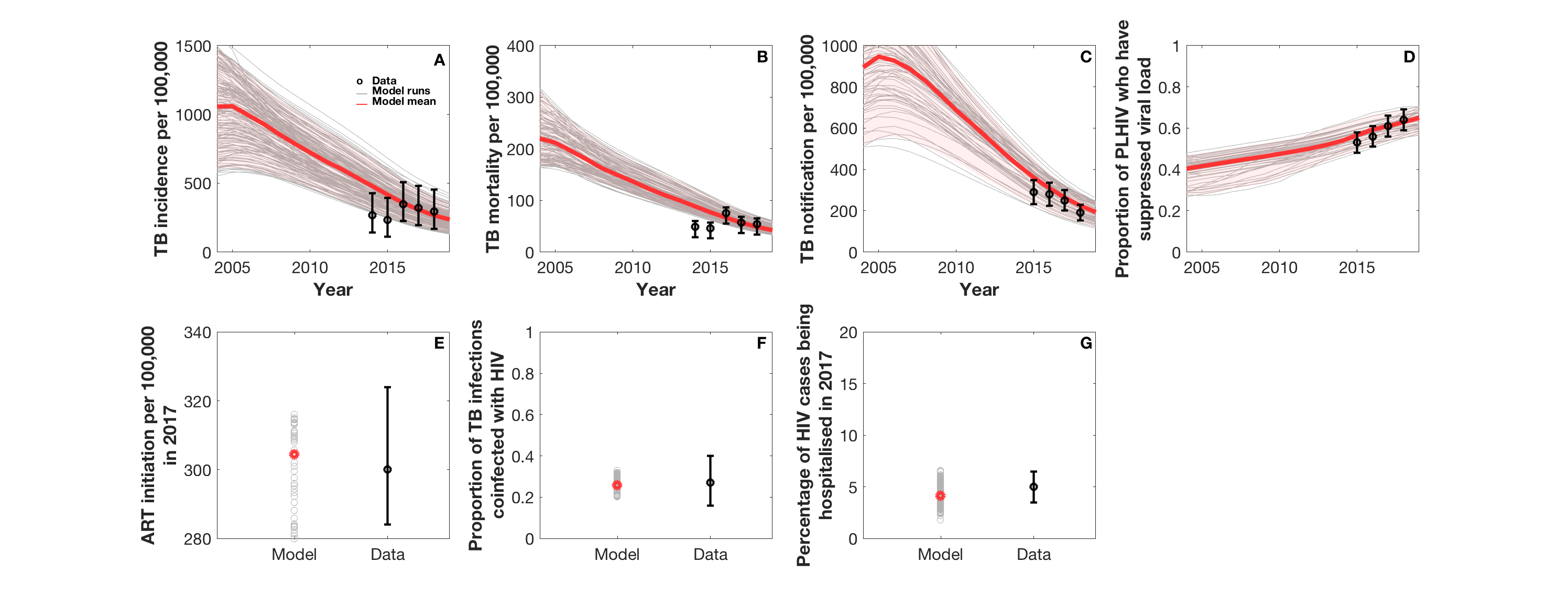

Supplement: S8 Fig — Data points are described in S2 Table. (A) TB incidence; (B) TB mortality per 100,000; (C) TB notifications per 100,000; (D) proportion of PLHIV with suppressed viral loads; (E) ART initiations per 100,000; (F) proportion of TB cases coinfected with HIV; (G) percentage of HIV cases being hospitalised annually. (TIF) [file pmed.1003466.s008.tif]

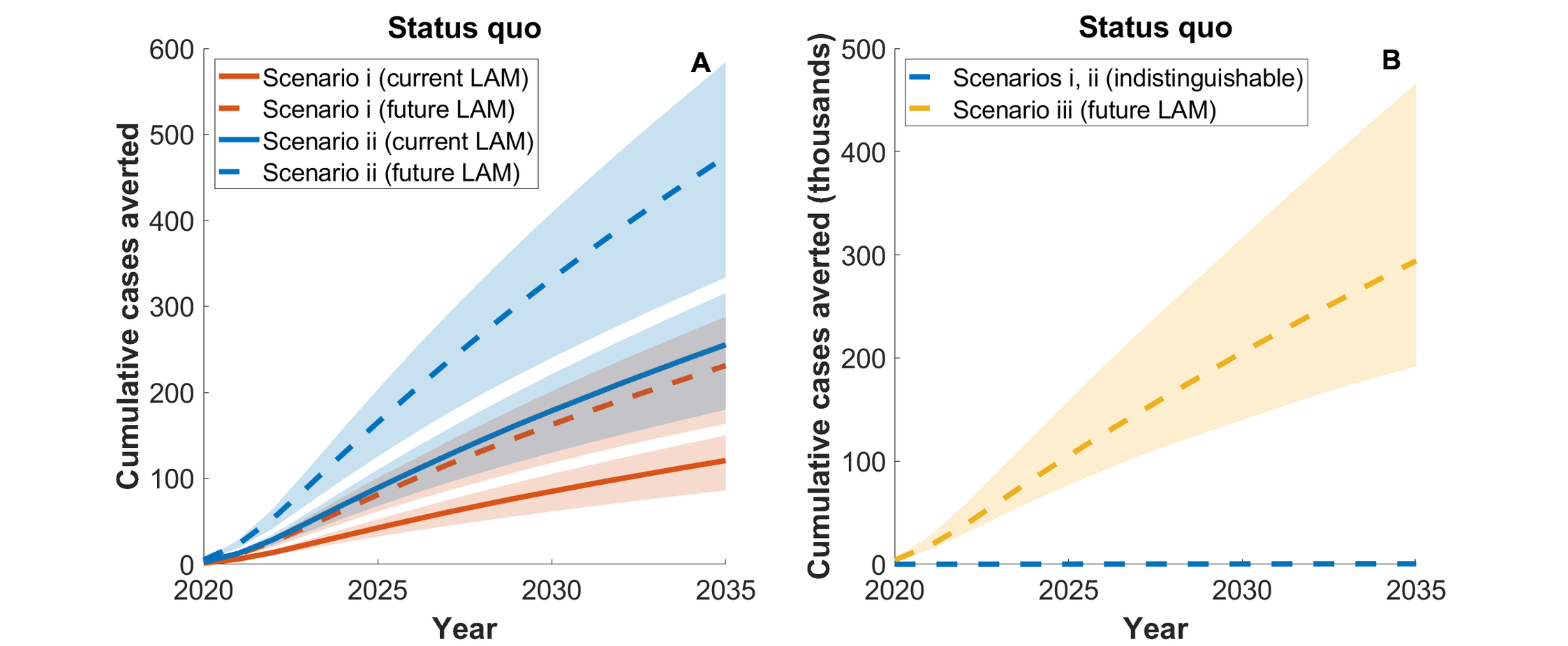

Supplement: S9 Fig — Under this comparator, the current standard of TB care in Kenya is assumed to continue indefinitely. Shaded areas show Bayesian 95% credible intervals. Solid lines depict a currently licensed test, while dashed lines depict a future LAM test. Colours represent different implementation scenarios: inpatients only (red, scenario i), plus outpatients (blue, scenario ii), and plus routine TB care (yellow, scenario iii). (A) depicts scenarios i and ii only, while (B) additionally shows scenario iii (shown separately owing to the change in scale). Cumulative impacts over the period 2020–2035 are summarised in S3 Table. (TIF) [file pmed.1003466.s009.tif]
