# Supplementary material for: The potential impact of urine-LAM diagnostics on tuberculosis incidence and mortality: A modelling analysis
Source: PLoS Med. 2020 Dec 11;17(12):e1003466. doi: 10.1371/journal.pmed.1003466 (PMC7732057; doi:10.1371/journal.pmed.1003466)
Supplement: S1 Table. TB incidence and mortality, and number of patients initiating ART per year, in inpatient and outpatient settings — (DOCX) [file pmed.1003466.s010.docx]

**S1 Table. TB incidence and mortality, and number of patients initiating ART per year, in inpatient and outpatient settings**

| Year | Number of ART initiations amongst outpatients per year | Number of ART initiations amongst inpatients per year |
| --- | --- | --- |
| 2020 | 363,470 (318,980-397,040) | 183,150 (141,590-234,830) |
| 2021 | 318,200 (278,680-347,300) | 165,250 (128,570-211,180) |
| 2022 | 283,580 (247,160-308,970) | 149,590 (116,720-190,860) |
| 2023 | 257,840 (223,960-281,260) | 137,570 (107,330-175,170) |
| 2024 | 239,320 (207,340-261,710) | 128,830 (100,350-163,620) |
| 2025 | 225,960 (195,400-247,790) | 122,210 (95,240-155,160) |
| 2026 | 216,220 (186,770-237,770) | 117,400 (91,490-148,970) |
| 2027 | 209,180 (180,470-230,450) | 113,950 (88,720-144,400) |
| 2028 | 203,720 (175,640-224,770) | 111,330 (86,580-140,860) |
| 2029 | 199,140 (171,620-219,930) | 109,090 (84,770-137,870) |
| 2030 | 194,900 (167,920-215,360) | 106,950 (83,080-135,090) |
| 2031 | 190,860 (164,420-210,930) | 104,860 (81,440-132,400) |
| 2032 | 186,800 (160,900-206,430) | 102,700 (79,770-129,670) |
| 2033 | 182,630 (157,310-201,790) | 100,460 (78,050-126,860) |
| 2034 | 178,320 (153,610-196,980) | 98,130 (76,250-123,930) |
| 2035 | 173,800 (149,720-191,920) | 95,660 (74,350-120,840) |
