# Supplementary material for: The potential impact of urine-LAM diagnostics on tuberculosis incidence and mortality: A modelling analysis
Source: PLoS Med. 2020 Dec 11;17(12):e1003466. doi: 10.1371/journal.pmed.1003466 (PMC7732057; doi:10.1371/journal.pmed.1003466)
Supplement: S2 Table — (DOCX) [file pmed.1003466.s011.docx]

**S2 Table. Calibration targets for Kenya used to estimate model parameters.**

| Indicator | | Value | Source |
| --- | --- | --- | --- |
| *TB epidemiology* | | | |
| TB incidence, 2018 | | 292 per 100,000 [179-432] | WHO (2019) [1] |
| Mortality, 2018 | HIV- | 38 per 100,000 [22-59] |  |
|  | HIV+ | 26 per 100,000 [16-38] |  |
| Notification rate, 2018 | | 189 per 100,000 [151-226] |  |
| *HIV epidemiology and care* | | | |
| HIV prevalence, 2017 | | 1.5 million  [1.3-1.7] | AIDSinfo [2] |
| Proportion of TB infections coinfected with HIV | | 0.27 [0.16-0.40] | WHO (2019) [1] |
| Proportion of PLHIV who have suppressed viral load | | 0.68 [0.60-0.79] | AIDSinfo [2] |

Additional data (including CD4 categories by HIV status, and percentage of HIV cases being hospitalised annually) are the same as for South Africa (Table 3).

**References**

[1] World Health Organization, ‘Global tuberculosis report 2019’, World Health Organization, 2019.

[2] UNAIDS, ‘AIDSinfo’. http://aidsinfo.unaids.org (accessed Oct. 08, 2019).
