# Supplementary material for: The potential impact of urine-LAM diagnostics on tuberculosis incidence and mortality: A modelling analysis
Source: PLoS Med. 2020 Dec 11;17(12):e1003466. doi: 10.1371/journal.pmed.1003466 (PMC7732057; doi:10.1371/journal.pmed.1003466)
Supplement: S3 Table — (DOCX) [file pmed.1003466.s012.docx]

**S3 Table. Projected cumulative impact relative to a ‘status quo’ comparator, Kenya.**

| Deployment level | LAM test | Incidence averted between 2020 - 2035 | | TB deaths averted between 2020 – 2035 | | TB deaths averted amongst inpatients between 2020-2035 | |
| --- | --- | --- | --- | --- | --- | --- | --- |
|  |  | Number | Percent | Number | Percent | Number | Percent |
| Inpatients, scenario (i) | **Currently licensed LAM test** | 121 (86-150) | 0.008 (0.006-0.011) | 49 (36-61) | 0.023 (0.019-0.029) | 14 (10-18) | 3.00 (2.77-3.47) |
|  | **Future LAM test** | 231 (163-288) | 0.015 (0.012-0.021) | 94 (68-116) | 0.044 (0.037-0.055) | 26 (19-34) | 5.71 (5.32-6.61) |
| Inpatients and Outpatients, scenario (ii) | **Currently licensed LAM test** | 255 (180-316) | 0.017 (0.013-0.024) | 96 (70-119) | 0.046 (0.038-0.057) | 14 (10-18) | 3.09 (2.85-3.59) |
|  | **Future LAM test** | 474 (333-584) | 0.031 (0.024-0.044) | 180 (130-223) | 0.085 (0.072-0.105) | 27 (20-35) | 5.88 (5.47-6.81) |
| Inpatients, outpatients and routine TB care, scenario (iii) | **Future LAM test** | 294,140 (191,930-466,480) | 19.8 (18.1-22.7) | 57,935 (38,818-86,317) | 27.9 (25.7-31.4) | 134 (98-204) | 30.7 (28.8-33.9) |

Under this comparator, we assume the current standard of TB care in Kenya to continue indefinitely.
