# Supplementary material for: The potential impact of urine-LAM diagnostics on tuberculosis incidence and mortality: A modelling analysis
Source: PLoS Med. 2020 Dec 11;17(12):e1003466. doi: 10.1371/journal.pmed.1003466 (PMC7732057; doi:10.1371/journal.pmed.1003466)
Supplement: S4 Table — (DOCX) [file pmed.1003466.s013.docx]

**S4 Table. Projected cumulative impact relative to an ‘Xpert scale-up’ comparator, Kenya.**

| Deployment level | LAM test | Incidence averted between 2020 - 2035 | | TB deaths averted between 2020 – 2035 | | TB deaths averted amongst inpatients between 2020-2035 | |
| --- | --- | --- | --- | --- | --- | --- | --- |
|  |  | Number | Percent | Number | Percent | Number | Percent |
| Inpatients, scenario (i) | **Currently licensed LAM test** | 28 (20-36) | 0.003 (0.002-0.004) | 22 (16-27) | 0.015 (0.013-0.019) | 8 (6-10) | 2.62 (2.41-3.05) |
|  | **Future LAM test** | 53 (37-69) | 0.005 (0.004-0.007) | 41 (31-51) | 0.029 (0.024-0.036) | 16 (12-19) | 5.02 (4.64-5.83) |
| Inpatients and Outpatients, scenario (ii) | **Currently licensed LAM test** | 55 (39-73) | 0.005 (0.004-0.007) | 40 (30-49) | 0.028 (0.024-0.035) | 8 (6-10) | 2.69 (2.47-3.14) |
|  | **Future LAM test** | 103 (73-135) | 0.009 (0.007-0.014) | 74 (56-93) | 0.053 (0.045-0.065) | 16 (12-20) | 5.14 (4.76-5.98) |
| Inpatients, outpatients and routine TB care, scenario (iii) | **Future LAM test** | 38,492 (26,220-51,489) | 3.51 (3.15-4.00) | 13,495 (9,659-17,827) | 9.63 (8.55-10.5) | 51 (39-67) | 17.0 (16.2-17.9) |

Under this comparator we assume comprehensive expansion of access to sputum Xpert across Kenya, such that all individuals with symptoms suggestive of TB are tested with Xpert on their first presentation for care. Numbers in the table show model-projected impact of LAM tests when deployed as an adjunct to Xpert, assuming (in all settings shown in the table) a simple diagnostic algorithm where TB is diagnosed if either LAM tests or Xpert is positive for TB. As described in the main text, in the model we also allow for clinical diagnosis amongst bacteriologically negative symptomatics.
