# Supplementary material for: The potential impact of urine-LAM diagnostics on tuberculosis incidence and mortality: A modelling analysis
Source: PLoS Med. 2020 Dec 11;17(12):e1003466. doi: 10.1371/journal.pmed.1003466 (PMC7732057; doi:10.1371/journal.pmed.1003466)
Supplement: S1 Text — (DOCX) [file pmed.1003466.s014.docx]

**S1 Text. Materials and methods**

Below, we describe the model, including its structure, governing equations and parameters. The model was constructed in MATLAB.

***1.1. Model structure***

*Overview*

We modelled TB transmission amongst adults (>15yo) in South Africa, incorporating the role of HIV in driving TB dynamics. We calibrated the model to TB incidence, mortality and notification rates. We also used HIV incidence, the proportion of PLHIV with and without TB, their CD4 cell counts, ART coverage and percentage of HIV cases being hospitalised annually as inputs (Table 3).

The model is stratified by pulmonary and extrapulmonary TB. Amongst those with HIV, the model is further stratified by CD4 count, using three categories: <100, 100 – 200, and >200, to align with the most recent WHO 2019 guidelines (Table 3) for the use of LF-LAM [1]. The model ignores drug resistance, as well as age structure in the population. However, it distinguishes different settings for TB and HIV care: routine TB services, those receiving HIV care in outpatient clinics, and those receiving HIV care as hospital inpatients.

*Routine TB services*

We assumed that those with active TB seek care at a certain per-capita rate, calibrated to epidemiological data (Table 2), and allowing for different careseeking rates by HIV-negative and -positive status. We assumed that each visit to a healthcare provider results in TB diagnosis with a probability governed by a combination of test sensitivity and clinical diagnosis. Amongst those failing to be diagnosed, we assumed temporary dropout from the care cascade, to seek care again after a given per-capita rate (again, calibrated to epidemiological data).

South Africa has recently undergone an expansion in the use of molecular diagnostics such as GeneXpert, for sputum-based diagnosis in routine TB services [2]. Under the introduction of LAM testing in routine TB services (scenario iii in the main text), we assumed that baseline levels of Xpert remain the same, but that LAM testing is used adjunctively, with a positive result on either test leading to a TB diagnosis. To incorporate clinical diagnosis, we assumed that 20% and 30% of negative test results are clinically diagnosed and offered treatment, amongst HIV-negative and HIV-positive patients, respectively [3]. The number of individuals initiating TB treatment, is defined by the following:

*Baseline, status quo comparator*:

$o_{R_{h}}=\varepsilon_{R}\kappa_{3}\kappa_{4}\phi_{R}\omega_{R}$ (1)

Where the proportion of TB cases diagnosed correctly per careseeking attempt, $o_{R_{h}}$, is calibrated to TB notification rates.

*Baseline, Xpert scale-up comparator:*

$$o_{R_{h}}={[1-(1-\varepsilon}_{R}\kappa_{3}\kappa_{4})(1-{\vartheta_{h}^{sputum}X_{sn})]\phi}_{R}\omega_{R}$$

 (2)

*Intervention, status quo comparator:*

$o_{R_{h}}={[1-(1-\varepsilon}_{R}\kappa_{3}\kappa_{4}){(1-\left( 1-\left( \varepsilon_{R}\kappa_{3}\kappa_{4}+\vartheta_{h}^{urine}F_{sn} \right) \right)\iota_{h})(1-\vartheta_{h}^{urine}F_{sn})]\phi}_{R}\omega_{R}$ (3)

*Intervention, Xpert scale-up comparator:*

$o_{R_{h}}={[1-(1-\varepsilon}_{R}\kappa_{3}\kappa_{4}){(1-\left( 1-\left( \varepsilon_{R}\kappa_{3}\kappa_{4}+\vartheta_{h}^{urine}F_{sn} \right) \right)\iota_{h})(1-\vartheta_{h}^{urine}F_{sn})(1-\vartheta_{h}^{sputum}X_{sn})]\phi}_{R}\omega_{R}$ (4)

*HIV Inpatients*

The model captures the hospitalisation of PLHIV, at a CD4-dependent rate, reflecting data from South Africa on HIV-associated inpatient admissions (Table 3, and S2 Fig). Upon admission, we assumed that of those with signs and symptoms of TB, a given proportion are offered an Xpert test at baseline. Under the introduction of LAM testing (scenarios i and ii in the main text), we assumed – consistent with current WHO guidelines [1] - that those with signs and symptoms of TB, and all those with CD4 cell count <200 cells/μl independent of symptoms, are offered a LAM test as an adjunctive test to Xpert, again allowing for clinical diagnosis amongst those with a negative test result.

*Baseline, status quo comparator*:

$o_{Ih}=\vartheta_{h}^{sputum}\xi_{I}X_{sn}s_{I}\omega_{I}\phi$ (5)

*Baseline, Xpert scale-up comparator:*

$o_{Ih}=[1-\left( 1-\vartheta_{h}^{sputum}\xi_{I}X_{sn} \right)\left( 1-\vartheta_{h}^{sputum}X_{sn} \right)]s_{I}\omega_{I}\phi$ (6)

*Intervention, status quo comparator, currently licensed LAM test:*

$o_{Ih}=[1-\left( 1-\vartheta_{h}^{sputum}\xi_{I}X_{sn} \right)\left( 1-((1-\vartheta_{h}^{sputum}\xi_{I}X_{sn}+\vartheta_{h}^{urine}A_{sn}))\iota_{h}) \right)(1-\vartheta_{h}^{urine}A_{sn})]s_{I}\omega_{I}\phi$ (7)

*Intervention, status quo comparator, future LAM test:*

$o_{Ih}=[1-\left( 1-\vartheta_{h}^{sputum}\xi_{I}X_{sn} \right)\left( 1-((1-\vartheta_{h}^{sputum}\xi_{I}X_{sn}+\vartheta_{h}^{urine}F_{sn}))\iota_{h}) \right)(1-\vartheta_{h}^{urine}F_{sn})]s_{I}\omega_{I}\phi$ (8)

*Intervention, Xpert scale-up comparator, currently licensed LAM test:*

$o_{Ih}=[1-\left( 1-\vartheta_{h}^{sputum}\xi_{I}X_{sn} \right)\left( 1-((1-(\vartheta_{h}^{sputum}\xi_{I}X_{sn}+\vartheta_{h}^{urine}A_{sn}))\iota_{h}) \right)(1-\vartheta_{h}^{urine}A_{sn})\left( 1-\vartheta_{h}^{sputum}X_{sn} \right)]s_{I}\omega_{I}\phi$ (9)

*Intervention, Xpert scale-up comparator, future LAM test:*

$o_{Ih}=[1-\left( 1-\vartheta_{h}^{sputum}\xi_{I}X_{sn} \right)\left( 1-((1-(\vartheta_{h}^{sputum}\xi_{I}X_{sn}+\vartheta_{h}^{urine}F_{sn}))\iota_{h}) \right)(1-\vartheta_{h}^{urine}F_{sn})\left( 1-\vartheta_{h}^{sputum}X_{sn} \right)]s_{I}\omega_{I}\phi$ (10)

*HIV Outpatients*

The model also captures the provision of HIV care in outpatient settings, assuming CD4-dependent rates of ART initiation in these settings. These rates were calibrated to match data from South Africa for the distribution of CD4 counts amongst those initiating ART in outpatient facilities (Table 3, and S2 Fig). Upon initiating HIV care, we assumed that of those with signs and symptoms of TB, a certain proportion are offered an Xpert test. As above, under the introduction of LAM testing (scenario ii in the main text), we assumed – consistent with current WHO guidelines [1] - that those with signs and symptoms of TB and those with a CD4 cell count <100 cells/μl independent of symptoms, are offered a LAM test as an adjunctive test to Xpert, again allowing for clinical diagnosis amongst those with a negative test result.

*Baseline, status quo comparator*:

$o_{Oh}=\vartheta_{h}^{sputum}\xi_{O}X_{sn}s_{O}\omega_{O}\phi$ (11)

*Baseline, Xpert scale-up comparator:*

$o_{Oh}=[1-\left( 1-\vartheta_{h}^{sputum}\xi_{O}X_{sn} \right)\left( 1-\vartheta_{h}^{sputum}X_{sn} \right)]s_{O}\omega_{O}\phi$ (12)

*Intervention, status quo comparator, currently licensed LAM test:*

$o_{Oh}=[1-\left( 1-\vartheta_{h}^{sputum}\xi_{O}X_{sn} \right)\left( 1-((1-(\vartheta_{h}^{sputum}\xi_{O}X_{sn}+\vartheta_{h}^{urine}A_{sn}))\iota_{h}) \right)(1-\vartheta_{h}^{urine}A_{sn})]s_{O}\omega_{O}\phi$(13)

*Intervention, status quo comparator, future LAM test:*

$o_{Oh}=[1-\left( 1-\vartheta_{h}^{sputum}\xi_{O}X_{sn} \right)\left( 1-((1-(\vartheta_{h}^{sputum}\xi_{O}X_{sn}+\vartheta_{h}^{urine}F_{sn}))\iota_{h}) \right)(1-\vartheta_{h}^{urine}F_{sn})]s_{O}\omega_{O}\phi$ (14)

*Intervention, Xpert scale-up comparator, currently licensed LAM test:*

$o_{Oh}=[1-\left( 1-\vartheta_{h}^{sputum}\xi_{O}X_{sn} \right)\left( 1-((1-(\vartheta_{h}^{sputum}\xi_{O}X_{sn}+\vartheta_{h}^{urine}A_{sn}))\iota_{h}) \right)(1-\vartheta_{h}^{urine}A_{sn})\left( 1-\vartheta_{h}^{sputum}X_{sn} \right)]s_{O}\omega_{O}\phi$ (15)

*Intervention, Xpert scale-up comparator, future LAM test:*

$o_{Oh}=[1-\left( 1-\vartheta_{h}^{sputum}\xi_{O}X_{sn} \right)\left( 1-((1-(\vartheta_{h}^{sputum}\xi_{O}X_{sn}+\vartheta_{h}^{urine}F_{sn}))\iota_{h}) \right)(1-\vartheta_{h}^{urine}F_{sn})\left( 1-\vartheta_{h}^{sputum}X_{sn} \right)]s_{O}\omega_{O}\phi$ (16)

***1.2. Governing equations for the mathematical transmission model***

The equations correspond to the model described in Fig 1. State variables (capital letters) are as listed in Table A in S1 Text, while model parameters (lower-case and Greek letters) are as listed in Table 2.

**Table A. Model stages and subscript description**

| **Model stage** | **Description** |
| --- | --- |
| ***U_h_*** | Uninfected |
| ***L_h_*** | Latent infection |
| ***I_h_*** | Active TB |
| ***Dx_h_*** | Sought care, awaiting TB diagnosis |
| ***Tx_h_*** | Undergoing TB treatment |
| ***E_h_*** | Between seeking care, after misdiagnosis or loss to follow up |
| ***R^TC^_h_*** | Recovered, after treatment completion, low risk of relapse |
| ***R^SC^_h_*** | Recovered, after self-cure, high risk of relapse |
| ***A_h_*** | Inpatient admission, awaiting TB diagnosis |
| ***Subscript*** | **Description** |
| ***h*** | HIV status (0 = HIV-, 1 = HIV+ >200, 2 = HIV+ 100-200, 3 = HIV+ <100 and 4 = ART+ virally suppressed), 5 = ART+ (not virally suppressed) >200, 6 = ART+ 100-200, 7 = HIV+ <100 |
| ***Superscript*** | **Description** |
| ***inp*** | Inpatient (hospitalised HIV-positive patients) |
| ***out*** | Outpatient |

*Uninfected* (17)

$\frac{dU_{h}(t)}{dt}=\left\{ \begin{aligned} T+W_{h}^{\left( 1 \right)}\left( t \right)-\left[ \lambda\left( t \right)+\mu_{1} \right]U_{h}\left( t \right) h=0 \\ \\ {W_{h}^{\left( 1 \right)}\left( t \right)+\psi}_{h}U_{h}^{inp}\left( t \right)-\left[ {\lambda\left( t \right)+\upsilon}_{h}+\mu_{1}+\mu_{3_{h}} \right]U_{h}\left( t \right) h>0 \end{aligned} \right.$

*Latency* (18)

$\frac{dL_{h}(t)}{dt}=\left\{ \begin{aligned} W_{h}^{\left( 2 \right)}\left( t \right)+\pi\lambda\left( t \right)\left[ 1-\theta_{h} \right]\left[ L_{h}\left( t \right)+R_{h}^{TC}\left( t \right)+R_{h}^{SC}\left( t \right) \right]+\lambda\left( t \right)\left[ 1-\theta_{h} \right]U_{h}\left( t \right) h=0 \\ -{[\rho}_{h}{+\mu_{1}]L}_{h}\left( t \right) \\ \\ W_{h}^{\left( 2 \right)}\left( t \right)+\psi_{h}L_{h}^{inp}\left( t \right)+ \pi\lambda\left( t \right)\left[ 1-\theta_{h} \right]\left[ L_{h}\left( t \right)+R_{h}^{TC}\left( t \right)+R_{h}^{SC}\left( t \right) \right] h>0 \\ +\lambda\left( t \right)\left[ 1-\theta_{h} \right]U_{h}\left( t \right)-{[\rho}_{h}{+\upsilon_{h}+\mu_{1}+\mu_{3_{h}}]L}_{h}\left( t \right) \end{aligned} \right.$

*Active disease, pre-careseeking* (19)

$\frac{dI_{h}(t)}{dt}=\left\{ \begin{aligned} W_{h}^{\left( 3 \right)}\left( t \right)+\theta_{h}\lambda\left( t \right)U_{h}\left( t \right)+\pi\theta_{h}\lambda\left( t \right)\left[ L_{h}\left( t \right)+R_{h}^{TC}\left( t \right)+R_{h}^{SC}\left( t \right) \right]+\rho_{h}L_{h}\left( t \right) h=0 \\ {+r}_{2}R_{h}^{TC}\left( t \right)+r_{1}R_{h}^{SC}\left( t \right)-\left[ {\delta_{1}+\varphi_{h}+\mu}_{1}+\mu_{2} \right]I_{h}\left( t \right) \\ \\ W_{h}^{\left( 3 \right)}\left( t \right)+\psi_{h}I_{h}^{inp}\left( t \right)+\theta_{h}\lambda\left( t \right)U_{h}\left( t \right)+\pi\theta_{h}\lambda\left( t \right)\left[ L_{h}\left( t \right)+R_{h}^{TC}\left( t \right)+R_{h}^{SC}\left( t \right) \right] h>0 \\ +\rho_{h}L_{h}\left( t \right)+r_{2}R_{h}^{TC}\left( t \right)+r_{1}R_{h}^{SC}\left( t \right)-[\delta_{1}\kappa_{2}{+\varphi_{h}+\varpi_{h}+\upsilon_{h}+\mu_{1}+\mu_{2}+\mu_{3_{h}}]I}_{h}\left( t \right) \end{aligned} \right.$

*Awaiting diagnosis* (20)

$\frac{d{Dx}_{h}(t)}{dt}=\left\{ \begin{aligned} W_{h}^{\left( 4 \right)}\left( t \right)+\delta_{1}I_{h}\left( t \right)+\delta_{2}E_{h}\left( t \right)-[\varphi_{h}+o_{R_{h}}+\mu_{1}+\mu_{2}]{Dx}_{h}\left( t \right) h=0 \\ \\ W_{h}^{\left( 4 \right)}\left( t \right)+\delta_{1}\kappa_{2}I_{h}\left( t \right)+\delta_{2}\kappa_{2}E_{h}\left( t \right) h>0 \\ -[\varphi_{h}+o_{R_{h}}+\mu_{1}+{\mu_{2}+ \mu}_{3\_h}+\varpi_{h}+{\upsilon_{h}]Dx}_{h}\left( t \right) \end{aligned} \right.$

*Treatment* (21)

$\frac{d{Tx}_{h}(t)}{dt}=\left\{ \begin{aligned} W_{h}^{\left( 5 \right)}\left( t \right)+o_{R_{h}}{Dx}_{h}\left( t \right)-\left[ \tau+\mu_{1} \right]{Tx}_{h}\left( t \right) h=0 \\ \\ W_{h}^{\left( 5 \right)}\left( t \right)+\psi_{h}{Tx}_{h}^{inp}\left( t \right)+o_{R_{h}}{Dx}_{h}\left( t \right)+o_{O_{h}}{Dx}_{h}^{out}\left( t \right) h>0 \\ -[\tau{+\upsilon_{h}+\mu_{1}+\mu_{3_{h}}]Tx}_{h}\left( t \right) \end{aligned} \right.$

*Missed diagnosis/initial loss to follow up* (22)

$\frac{dE_{h}(t)}{dt}=\left\{ \begin{aligned} W_{h}^{\left( 6 \right)}\left( t \right)+[1-o_{R_{h}}]{Dx}_{h}\left( t \right)+\tau[1-\chi_{h}]{Tx}_{h}\left( t \right)-[\delta_{2}+\varphi_{h}{+\mu_{1}+\mu_{2}]E}_{h}\left( t \right) h=0 \\ \\ {W_{h}^{\left( 6 \right)}\left( t \right)+\psi}_{h}E_{h}^{inp}\left( t \right)+\left[ 1-o_{R_{h}} \right]{Dx}_{h}\left( t \right)+\left[ 1-o_{O_{h}} \right]{Dx}_{h}^{out}\left( t \right) h>0 \\ + \tau[1-\chi_{h}]{Tx}_{h}(t)-[\delta_{2}+\varphi_{h}+\varpi_{h}+\upsilon_{h}+\mu_{1}+{\mu_{2}+\mu}_{3\_h}]E_{h}\left( t \right) \end{aligned} \right.$

*Post-treatment recovery*  (23)

$\frac{dR_{h}^{TC}(t)}{dt}=\left\{ \begin{aligned} W_{h}^{\left( 7 \right)}\left( t \right)+\tau\chi_{h}{Tx}_{h}\left( t \right)+\varsigma R_{h}^{SC}\left( t \right)-\left[ r_{2}+\mu_{1} \right]R_{h}^{TC}\left( t \right) h=0 \\ \\ {W_{h}^{\left( 7 \right)}\left( t \right)+\psi}_{h}R_{h}^{TC_{inp}}\left( t \right)+\tau\chi_{h}{Tx}_{h}\left( t \right)+\varsigma R_{h}^{SC_{inp}}\left( t \right) h>0 \\ -[r_{2}+ \upsilon_{h}+\mu_{1}+\mu_{3\_h}]R_{h}^{TC}\left( t \right) \end{aligned} \right.$

*Self-cure* (24)

$\frac{dR_{h}^{SC}(t)}{dt}=\left\{ \begin{aligned} W_{h}^{\left( 8 \right)}\left( t \right)+\varphi_{h}\left[ E_{h}\left( t \right)+{Dx}_{h}\left( t \right)+I_{h}\left( t \right) \right]-\left[ \varsigma+r_{1}+\mu_{1} \right]R_{h}^{SC}\left( t \right) h=0 \\ \\ {W_{h}^{\left( 8 \right)}\left( t \right)+\psi}_{h}R_{h}^{SC\_inp}\left( t \right)+\varphi_{h}\left[ E_{h}\left( t \right)+{Dx}_{h}\left( t \right)+I_{h}\left( t \right) \right] h>0 \\ -[\varsigma+r_{1}+\upsilon_{h}+\mu_{1}+\mu_{3\_h}]R_{h}^{SC\_inp}\left( t \right) \end{aligned} \right.$

*Uninfected, inpatients* (25)

$\frac{dU_{h}^{inp}(t)}{dt}={W_{h}^{\left( 9 \right)}\left( t \right)+\upsilon}_{h}U_{h}\left( t \right){- [\lambda\left( t \right)+\psi}_{h}+\mu_{1}+\mu_{3\_h}]U_{h}^{inp}\left( t \right) h>4$

*Latency, inpatients* (26)

$$\frac{dL_{h}^{inp}\left( t \right)}{dt}=W_{h}^{\left( 10 \right)}\left( t \right)+\upsilon_{h}L_{h}\left( t \right)+\lambda\left( t \right)[{1-\theta}_{h}]U_{h}^{inp}\left( t \right)+\pi\lambda\left( t \right)[1-\theta_{h}][L_{h}^{inp}\left( t \right) h>4$$

$+R_{h}^{TC\_inp}\left( t \right)+R_{h}^{SC\_inp}\left( t \right)]{- [\psi}_{h}+\rho_{h}+\mu_{1}+\mu_{3\_h}]L_{h}^{inp}\left( t \right)$

*Active disease, inpatients*  (27)

$$\frac{dI_{h}^{inp}\left( t \right)}{dt}=W_{h}^{\left( 11 \right)}\left( t \right)+\rho_{h}L_{h}^{inp}\left( t \right)+\theta_{h}\lambda\left( t \right)U_{h}^{inp}\left( t \right)+r_{1}R_{h}^{SC_{inp}}\left( t \right)+r_{2}R_{h}^{TC_{inp}}\left( t \right) h>4$$

$+\pi\theta_{h}\lambda\left( t \right)\left[ L_{h}^{inp}\left( t \right)+R_{h}^{TC\_inp}\left( t \right)+R_{h}^{SC\_inp}\left( t \right) \right]-{[\varphi}_{h}+\psi_{h}+\mu_{1}+\mu_{2}+\mu_{3\_h}+\mu_{4}]I_{h}^{inp}(t)$

*Hospital admissions awaiting diagnosis, inpatients* (28)

$\frac{dA_{h}^{inp}(t)}{dt}={W_{h}^{\left( 12 \right)}\left( t \right)+\upsilon}_{h}[I_{h}\left( t \right)+{Dx}_{h}\left( t \right)+E_{h}\left( t \right)]-{[o}_{I_{h}}+\varphi_{h}+\mu_{1}+\mu_{2}{+\mu}_{3_{h}}+\mu_{4}]A_{h}^{inp}(t) h>4$

*TB treatment, inpatients* (29)

$$\frac{d{Tx}_{h}^{inp}(t)}{dt}={W_{h}^{\left( 13 \right)}\left( t \right)+\upsilon}_{h}{Tx}_{h}\left( t \right)+o_{I_{h}}A_{h}^{inp}\left( t \right)-\left[ \tau+\psi_{h}+\mu_{1}+\mu_{3_{h}} \right]{Tx}_{h}^{inp}\left( t \right) h>4$$

*Missed diagnosis/initial loss to follow up, inpatients* (30)

$$\frac{dE_{h}^{inp}\left( t \right)}{dt}=W_{h}^{\left( 14 \right)}\left( t \right)+\left[ 1-o_{I_{h}} \right]A_{h}^{inp}\left( t \right)+\tau\left[ 1-\chi_{h} \right]{Tx}_{h}^{inp}\left( t \right) h>4$$

$-{[\varphi}_{h}+\psi_{h}+\mu_{1}+{\mu_{2}+\mu}_{3_{h}}+\mu_{4}]E_{h}^{inp}(t)$

*Post-TB treatment recovery, inpatients* (31)

$$\frac{dR_{h}^{TC_{inp}}\left( t \right)}{dt}={W_{h}^{\left( 15 \right)}\left( t \right)+\tau\chi_{h}{Tx}_{h}^{inp}\left( t \right)+\upsilon}_{h}R_{h}^{TC}\left( t \right)+\varsigma R_{h}^{SC_{inp}}\left( t \right) h>4$$

$-{[\psi}_{h}+r_{2}+\mu_{1}+\mu_{3_{h}}]R_{h}^{TC_{inp}}(t)$

*Self-cure, inpatients* (32)

$$\frac{dR_{h}^{SC_{inp}}\left( t \right)}{dt}={W_{h}^{\left( 16 \right)}\left( t \right){+ \upsilon}_{h}R_{h}^{SC}\left( t \right)+\varphi}_{h}\left[ A_{h}^{inp}\left( t \right)+E_{h}^{inp}\left( t \right)+I_{h}^{inp}\left( t \right) \right]-[\varsigma+r_{1}+\psi_{h} h>4$$

$+\mu_{1}+\mu_{3\_h}]R_{h}^{SC\_inp}(t)$

*Awaiting diagnosis at ART initiation, outpatients* (33)

$\frac{d{Dx}_{h}^{out}(t)}{dt}={W_{h}^{\left( 17 \right)}\left( t \right)+\varpi}_{h}\left[ I_{h}\left( t \right)+{Dx}_{h}\left( t \right)+E_{h}\left( t \right) \right]-[{o_{O_{h}}+\mu}_{1}+{\mu_{2}+\mu}_{3_{h}}]{Dx}_{h}^{out}(t) h>4$

where term $T$ represents births into the uninfected TB compartment. We assume it is equivalent to the number of deaths occurring in our model to ensure a constant population size.

*Force of infection*

$\lambda\left( t \right)=\frac{Ϝ(t)}{N(t)}$ (34)

where

$Ϝ(t)=\beta\{\sum_{h=0} [I(t)+Dx(t)+E(t)]\}+\beta\kappa_{1}\{\sum_{h=1}^{4} [I(t)+I_{inp}(t)+Dx(t)+{Dx}_{out}(t)+E(t)]\},$ (35)

and N(t) is the total population at time *t*.

*HIV-transitions*

where terms in $W_{h}^{x}(t)$ represent transitions between HIV stages:

**Table B. HIV transitions**

| **HIV state** | **Symbol** |
| --- | --- |
| HIV- | h=0 |
| HIV+ >200 | h=1 |
| HIV+ 100-200 | h=2 |
| HIV+ <100 | h=3 |
| ART+ virally suppressed | h=4 |
| ART+ >200 (not virally suppressed) | h=5 |
| ART+ 100-200 (not virally suppressed) | h=6 |
| ART+ <100 (not virally suppressed) | h=7 |

We write $X_{h}^{z}(t)$ to denote HIV compartments. Super index *z* represents TB model stages described in the equations above (e.g. *z*=1 for the *uninfected stage*)

(36)

$$W_{h}^{z}=\left\{ \begin{aligned} -\gamma X_{h}^{z}\left( t \right) if h=0 \\ \gamma X_{0}^{z}\left( t \right) -\eta_{1}X_{h}^{z}\left( t \right)-\upsilon_{h}X_{h}^{z}\left( t \right)-\varpi_{h}X_{h}^{z}\left( t \right) if h=1 \\ \eta_{1}X_{1}^{z}\left( t \right) -\eta_{2}X_{h}^{z} \left( t \right)-\upsilon_{h}X_{h}^{z}\left( t \right)-\varpi_{h}X_{h}^{z}\left( t \right) if h=2 \\ \eta_{2}X_{2}^{z}(t) -\upsilon_{h}X_{h}^{z}(t) -\varpi_{h}X_{h}^{z}(t) if h=3 \\ \varpi_{h}\varrho X_{1}^{z}\left( t \right)+\upsilon_{h}\varrho X_{1}^{z}\left( t \right)+\varpi_{h}\varrho X_{2}^{z}\left( t \right)+\upsilon_{h}\varrho X_{2}^{z}\left( t \right)+\varpi_{h}\varrho X_{3}^{z}\left( t \right)+\upsilon_{h}\varrho X_{3}^{z}\left( t \right) if h=4 \\ \varpi_{h}\left( 1-\varrho\right)X_{1}^{z}\left( t \right)+\upsilon_{h}\left( 1-\varrho\right)X_{1}^{z}\left( t \right)-\eta_{1}X_{h}^{z}\left( t \right) if h=5 \\ \varpi_{h}\left( 1-\varrho\right)X_{2}^{z}\left( t \right)+\upsilon_{h}\left( 1-\varrho\right)X_{2}^{z}\left( t \right)+\eta_{1}X_{5}^{z}\left( t \right)-\eta_{2}X_{h}^{z} \left( t \right) if h=6 \\ \varpi_{h}\left( 1-\varrho\right)X_{3}^{z}\left( t \right)+\upsilon_{h}\left( 1-\varrho\right)X_{3}^{z}\left( t \right)+\eta_{2}X_{6}^{z}\left( t \right) if h=7 \end{aligned} \right.$$

***1.3. Model calibration***

We incorporated uncertainty in data and model inputs (Table 2, Table 3 and S2 Table) using an Adaptive Bayesian Markov Chain Monte Carlo (MCMC) from Haario et al [4].

We define the posterior distribution as,

$p\left( \theta| D \right)\propto L\left( D | \theta\right) . \pi\left( \theta\right)$ (37)

Where, θ is a vector of our data and model inputs subject to uncertainty, *L* is the likelihood of our data given θ (see below for its construction), and π is the prior distribution. We assumed uniform distributions (ranges shown in Table 2) for the prior distributions*.* We defined the likelihood by fitting beta and log-normal distributions to the calibration targets (Table 3) for all data that were proportions and for TB incidence, respectively. For example, the likelihood function for total TB incidence ($L_{{Inc}_{total}}$) can be written as:

$L_{{Inc}_{total}}\left( {Inc}_{total}\left( \theta\right) \right)$ (38)

Then, for a given set of parameters, $\theta$, the overall likelihood, $p\left( \theta\right)$, was calculated as follows:

$\log\left( p\left( \theta\right) \right)=\log\left( L_{{Inc}_{total}}\left( {Inc}_{total}\left( \theta\right) \right)+L_{x_{2}}\left( x_{2}\left( \theta\right) \right)+\ldots L_{x_{z}}(x_{z}\left( \theta\right)) \right)$ (39)

where ${'x}_{2},x_{3},\ldots^{'}$represent successive data elements in table 2.

We sampled from the posterior distribution using MCMC, which should provide an unbiased sample that approximates the posterior distribution. A sample is randomly selected from $p\left( \theta| D \right)$, which is then accepted or rejected, depending on the proposal distribution. We did this using Adaptive MCMC, using the approach first proposed by Haario et al. In brief, we modelled the proposal distribution using a multivariate normal distribution, with mean zero and a given covariance matrix $\Sigma$. Standard MCMC approaches require that $\Sigma$ be ‘tuned’ in order to sample efficiently from the posterior density, but in the present work, manual tuning is infeasible with the 122-dimensional parameter space being sampled from (Table 2). Instead, the Haario algorithm approximates the covariance matrix empirically by computing the covariance of already-sampled parameters, during the execution of MCMC. We assumed uniform priors for all model parameters: if the width of the uniform distribution for parameter $i$ is $w_{i}$, we initiated $\Sigma$ simply as a diagonal matrix, with diagonal terms $\left( \frac{w_{i}}{50} \right)^{2}$. We found that no further ‘tuning’ was required. We removed the burn-in period (10,000 iterations) once the MCMC had converged and selected every 50th sample to reduce any autocorrelation. We ended up with 5000 samples from the posterior distribution. By incorporating uncertainty in data and model inputs, we were able to propagate this uncertainty into our model projections.

**References**

[1] World Health Organisation, ‘Lateral flow urine lipoarabinomannan assay (LF-LAM) for the diagnosis of active tuberculosis in people living with HIV.’, World Health Organisation, 2019. Accessed: Jan. 13, 2020. [Online]. Available: https://www.who.int/tb/publications/2019/LAMPolicyUpdate2019/en/.

[2] Z. Qin, M. Pai, W. Van Gemert, S. Sahu, M. Ghiasi, and J. Creswell, ‘How is Xpert MTB/RIF being implemented in 22 high tuberculosis burden countries?’, *Eur. Respir. J.*, vol. 45, pp. 549–554, 2015.

[3] P. Naidoo *et al.*, ‘The South African tuberculosis care cascade: estimated losses and methodological challenges.’, *J. Infect. Dis.*, vol. 216, no. Suppl 7, pp. S702-713, 2017.

[4] H. Haario, E. Saksman, and J. Tamminen, ‘An adaptive metropolis algorithm.’, *Bernoulli*, vol. 7, no. 2, pp. 223–242, 2001.
